# Pan-cancer bone metastasis atlas at single-cell resolution identifies a distinct tumor-associated macrophage subset for mediating Denosumab-induced immunosensitization in lung cancer bone metastasis

**DOI:** 10.7150/ijbs.119777

**Published:** 2026-01-01

**Authors:** Xianglin Hu, Nan Du, Yansha Song, Ke Lang, Wanning Tong, Qingrong Ye, Xuesi Liu, Haoyu Zheng, Mo Cheng, Yingzheng Ji, Haibo Wu, Minghe Zhang, Xinhong He, Yan Zhang, Xiaomeng Li, Yao Zhu, Kun Li, Weiluo Cai, Wangjun Yan, Wending Huang

**Affiliations:** 1 Department of Musculoskeletal Oncology, Fudan University Shanghai Cancer Center, Shanghai, China.; 2 Department of Oncology, Shanghai Medical College, Fudan University, Shanghai, China.; 3 Department of Interventional Radiology, Fudan University Shanghai Cancer Center, Shanghai, China.; 4 Department of Pulmonary Medicine, Zhongshan Hospital, Fudan University, Shanghai, China.; 5 Department of Respiratory and Critical Care Medicine, Naval Medical Center of People's Liberation Army, Naval Medical University, Shanghai, China.; 6 Department of Orthopedics, Naval Medical Center of People's Liberation Army, Naval Medical University, Shanghai, China.; 7 Department of Thoracic Surgery, Naval Medical Center of People's Liberation Army, Naval Medical University, Shanghai, China.; 8 Department of Orthopedics, Gongli Hospital of Shanghai Pudong New Area, Shanghai, China.; 9 Department of Urology, Fudan University Shanghai Cancer Center, Shanghai, China.; 10 Health Science Center, East China Normal University, Shanghai, China.

**Keywords:** bone metastasis, Denosumab, tumor-associated macrophages, RANKL, single-cell sequencing

## Abstract

Lung cancer (LC), prostate cancer (PC), and breast cancer (BC) are the three most prevalent cancers that lead to bone metastasis (BoM). In this study, we conducted an integrated analysis of single-cell transcriptomic data from the primary tumors and BoM across PC, LC, and BC. We discover a novel subtype of tumor-associated macrophages (TAMs) that are positive both for matrix metalloproteinase 19 (MMP19) and receptor activator of nuclear factor-κB (RANK) expression (MMP19^+^ RANK^+^ TAMs). MMP19^+^ RANK^+^ TAMs demonstrate an increased level of M2 polarization and act as a critical driving factor for LC-BoM. MMP19^+^ RANK^+^ TAMs are organized in a ring-like arrangement surrounding the tumor nests, constructing a barrier structure that impedes the infiltration of CD8^+^ T cells into the tumor core in LC-BoM. RANKL inhibitor Denosumab has been shown to effectively reduce the level of M2 polarization, decrease the population of MMP19^+^ RANK^+^ TAMs, and disrupt their barrier structure. Denosumab facilitates the infiltration of CD8^+^ T cells into the interior of LC-BoM tissues. Based on this mechanism, we observed in both clinical cohorts and preclinical models that RANKL inhibitor can enhance the efficacy of immunotherapy in treating LC-BoM.

## 1. Introduction

Bone metastasis (BoM) is a frequent distant dissemination site for advanced malignant solid tumors. The vertebral column is the most common anatomical location of BoM. A population-based study including more than 2 million cancer patients reported that about 4.85% of cancer patients present BoM at the initial diagnosis 1. Lung cancer (LC), prostate cancer (PC), and breast cancer (BC) rank as the top 3 most common bone metastatic cancers. The proportion of LC-BoM accounts for 49.37%, PC-BoM accounts for 15.02%, and BC-BoM accounts for 13.57% among all cancer BoM cases. The five-year survival rate is significantly low in LC-BoM (8%), while relatively higher in PC-BoM (32%) and BC-BoM (41%) 1. More than 85% of LC-BoM cases are attributed to non-small cell lung cancer (NSCLC), primarily comprising lung adenocarcinoma (LUAD), lung squamous cell carcinoma (LUSC), and large cell cancers.

The bone microenvironment offers an optimal niche that facilitates the colonization and proliferation of circulating disseminated tumor cells. The bone metastatic microenvironment is composed of a diverse array of cellular and structural elements, including cancer cells, mononuclear macrophages, osteoclasts, immune cells, vascular endothelial cells, and the extracellular matrix 2. The BoM from various cancer types exhibits significant heterogeneity. From a phenotypic perspective, PC-BoM is predominantly osteogenic, whereas LC-BoM and BC-BoM exhibit osteolytic or mixed characteristics. Furthermore, the mechanisms underlying BoM formation and progression substantially differ depending on the primary cancer types. The PC cells within the bone marrow secrete osteocalcin, osteopontin, and bone morphogenic proteins (BMPs) to induce osteosclerosis 3. The BC cells within the bone microenvironment possess the capability to recruit and activate osteoclasts, which in turn secrete transforming growth factor-beta (TGF-β), insulin-like growth factor-1 (IGF-1), and interleukin-6 (IL-6). These factors facilitate the proliferation of BC cells and promote the development of macrometastasis 3.

Comparing the tumor microenvironment of BoM with that of primary tumors could facilitate the identification of key cellular components and signaling pathways implicated in BoM initiation. In this study, our objective is to compare the tumor microenvironment of BoM with that of primary tumors at a single-cell resolution in the three most prevalent cancers associated with BoM. Given that LC-BoM constitutes the largest patient population with BoM and is associated with the poorest prognosis, this study also aims to explore potential combination therapies to enhance the responsiveness of LC-BoM to immunotherapy.

## 2. Materials and Methods

### 2.1 scRNA-seq data acquisition, quality control and dimensionality reduction

We retrieved the scRNA-seq data from 9 PC tissue samples with 9 PC-BoM tissue samples; 3 LC tissue samples with 3 LC-BoM tissue samples; and 2 BC tissue samples with 2 BC-BoM tissue samples from the NCBI Gene Expression Omnibus (GEO) database. The GSM number for each sample was provided (**Table S1**). All scRNA-seq data were generated and sequenced using the 10× Genomics platform. The data were processed using the Cell Ranger (version 6.0.0) provided by 10x Genomics, and statistical summaries of the raw data were obtained. We utilized the R package Seurat (version 4.1.1) for data filtering 4. The specific filtering criteria were as follows: (1) Genes expressed in fewer than three cells were excluded; (2) Cells with a total gene count exceeding 5000 or falling below 400 were removed; (3) Cells with a mitochondrial gene proportion greater than 20% were also excluded. Furthermore, the detection and removal of doublets were performed using DoubletFinder (version 2.0.3). Next, multiple sample datasets were integrated, followed by cell clustering and subpopulation analysis using the Seurat software package (version 4.1.1). The detailed procedure was outlined as follows: (1) Standardization of gene expression levels was performed using the LogNormalize method within the "Normalization" function; (2) Principal component analysis (PCA) was conducted to reduce dimensionality; (3) Clustering analysis was executed on the standardized data utilizing the Louvain algorithm; (4) Dimensionality reduction and visualization of the data were achieved through the Uniform Manifold Approximation and Projection (UMAP) method.

### 2.2 Cell type annotation

We analyzed the differentially expressed genes between different clusters based on the Wilcox algorithm. The specific screening criteria were as follows: (1) The expression proportion of the gene in the cell population, pct.1 or pct.2 > 0.25; (2) avg_log2FC > 0.25; and (3) P value < 0.01. For the cell type annotation of each cluster, we investigated the expression levels of canonical markers reported in the literature. The expression profiles of markers for each cell type were visualized using FeaturePlot and violin plots generated via the Seurat FeaturePlot and VlnPlot functions.

### 2.3 Kyoto Encyclopedia of Genes and Genomes (KEGG) analysis

To reveal the potential functions of DEGs, KEGG enrichment analysis was performed using the "clusterProfiler" R package 5. Pathways with an adjusted p-value (p_adj) less than 0.05 were considered significantly enriched. Combining research interests with a background in biology, pathways related to cancer metastasis were selected and presented.

### 2.4 Pseudotime trajectory analysis

To map the differentiation or conversion of specific cell types, pseudotime trajectory analysis was conducted using Monocle2 (V 2.30.0) 6. First, within the Monocle2 framework, the dispersionTable function was employed to identify highly variable genes. Subsequently, dimensionality reduction was conducted using DDRTree, a method integrated into Monocle2. Finally, the trajectory was visualized via the plot_cell_trajectory function in Monocle2, allowing for the observation and analysis of the differentiation or conversion processes of specific cell types.

### 2.5 Switch gene analysis

The R package GeneSwitches (V0.1.0) 7 was utilized for switch gene analysis. The analysis process was based on the pseudo-temporal results of the Monocle2 to calculate the genes that became silent or activated during the differentiation process. Firstly, the genes in the differentiation trajectory were binarized to screen out potential switch genes with switch states. Then, the software conducted logistic regression analysis and McFadden's Pseudo R^2^ pseudo-temporal correlation analysis on these genes. Finally, the top switch genes were sorted and visualized on the pseudo-temporal sequence according to their switch times to demonstrate the key gene functions.

### 2.6 Transcription factor (TF) analysis

We used the Python-based pipeline software package pySCENIC (V 0.12.1) 8 to conduct transcription factor analysis on single-cell data. Firstly, the single-cell gene expression matrix and the list of transcription factors (v10nr_clust) were read. Then, we inferred the co-expression modules of TFs and candidate target genes through the pyscenic grn function (based on the GRNBoost2 algorithm) to form the initial regulons. Next, we utilized the pyscenic ctx function to perform cisTarget analysis on the initial regulons, screening for regulons. Finally, we calculated the activity intensity (AUC value) of each regulon in each cell using the AUCell algorithm (pyscenic aucell function), generating a cell-regulon activity matrix to quantify the heterogeneity of transcriptional regulation at the single-cell level.

### 2.7 Copy number variations (CNVs) analysis

We utilized inferCNV (v1.14.2) to infer CNVs in tumor cells 9. The gene expression matrix was extracted from single-cell RNA sequencing data (Seurat object). Thereafter, T cells were selected as the reference baseline. CNVs in genomic regions were identified by quantifying the relative deviation of gene expression between tumor cells and reference cells following standardization.

### 2.8 Intra-tumor heterogeneity (ITH) score analysis

We utilized a method based on principal component analysis (PCA) to quantitatively evaluate the ITH Score 10. Firstly, PCA was used to map the complex gene expression data of tumor cells into a low-dimensional space. In this low-dimensional space, the distribution characteristics of cells can more intuitively reflect their heterogeneity differences, and those cell groups with greater heterogeneity tend to be farther apart in space. Distance scores were assigned to each cell. These scores were integrated to achieve the quantification of the ITH Score. This quantification approach enables a more precise assessment of tumor cell heterogeneity, thereby providing robust data support for further investigations into the biological properties, treatment responses, and prognostic implications of tumors 10.

### 2.9 Tumor stemness (cytoTRACE) score

We used the cytoTRACE (v0.3.3) algorithm to calculate the tumor stemness score 11. Specifically, we took the annotated RData dataset as the analysis object and used the CytoTRACE function with default parameter settings to quantitatively evaluate the stemness characteristics of cells. Cells with high scores demonstrated stronger stemness features. These cells with high stemness features are highly likely to play a key role in the occurrence, development, metastasis, and drug resistance of tumors.

### 2.10 M2 polarization score analysis

We employed AUCell 12 (version: V1.24.0) to evaluate the M2 polarization state of macrophages. Using the AUCell algorithm, genes within each cell were ranked in descending order based on their expression levels. Subsequently, the cumulative distribution of genes in the target gene set was calculated from the sorted list, and the area under the curve (AUC) was determined to quantify the activity of the M2 gene set at the single-cell level.

### 2.11 Combination analysis and validation in Gene Expression Profiling Interactive Analysis 2 (GEPIA2)

GEPIA2 (http://gepia2.cancer-pku.cn/#index) is an updated version of GEPIA designed for analyzing RNA sequencing expression data from 9,736 tumor samples and 8,587 normal samples derived from the TCGA and GTEx projects, utilizing a standardized processing pipeline 13. GEPIA2 offers a range of customizable functionalities, including tumor/normal differential expression analysis, profiling by cancer type, patient survival analysis, and correlation analysis. The markers selected for CD8^+^ Tex were HAVCR2, TIGIT, LAG3, PDCD1, CXCL13, LAYN, and CD8. The markers selected for CD8^+^ Tcm were CCR7, SELL, IL7R, and CD8. The markers selected for CD8^+^ Tem were PDCD1, DUSP4, GZMK, GZMA, IFNG, and CD8. The cut-off values for high and low expression were set at the 75th and 25th percentiles, respectively.

### 2.12 Cell-cell interaction analysis

CellChat 14 was a computational tool for analyzing intercellular interactions, leveraging the CellChat R package to investigate ligand-receptor interactions within specific signaling pathways. CellChat objects were generated via the R package's processing pipeline, with cell information incorporated into the metadata slots of the object. A ligand-receptor interaction database was constructed, followed by receptor matching inference computations. Graphical visualization parameters were configured with nPatterns set to 5.

### 2.13 Multiple immune fluorescence (mIF) staining in conjunction with the tartrate-resistant acid phosphase (TRAP) staining

For a same paraffin-embedded tissue sample, mIF staining analysis was conducted on myeloid-lineage and T cells in two consecutive sections, respectively, to evaluate their spatial relationship. CHI3L1 Rabbit pAb (Bioss, bs-10215R) was diluted at a ratio of 1:100. MMP19 Rabbit pAb (Bioss, bs-10058R) was diluted at a ratio of 1:300. RANK Rabbit pAb (Bioss, bs-2695R) was diluted at a ratio of 1:200. CD163 (Abcam, ab182422) was diluted at a ratio of 1:300. CD68 (Biolynx, BX50031) was diluted at a ratio of 1:2000. Cytokeratin in Pan (panCK) (Biolynx, BX50143-C3) was diluted at a ratio of 1:1000. CCR7 Rabbit mAb (Zenbio, R380950) was diluted at a ratio of 1:10000. CD45RO (CST, 55618S) mouse mAb was diluted at a ratio of 1:1000. Mouse monoclonal antiPD-1 antibody (sino biological, 10377-MM23) was diluted at a ratio of 1:400. CD8 (Biolynx, BX50036) was diluted at a ratio of 1:300. TRAP is a specific marker enzyme of osteoclasts. TRAP staining is a staining method used to detect characteristic substances in bone tissue and bone cells, which makes osteoclasts appear wine red and their nuclei blue, thereby showing the distribution and quantity changes of osteoclasts. Fluorescence imaging scanner (3DHISTECH, Pannoramic 2.2.0 MIDI) was used for panoramic scanning. The Halo analysis software (provided by Indica Lab, Version 3.2.1851.229) was utilized for the analysis of expression patterns and quantitative evaluation.

### 2.14 Real-world clinical patient cohorts

The clinical cohort study had obtained ethical approval from FUSCC. The clinical cohort 1 retrospectively included 814 PC patients, 121 LC patients and 66 BC patients (**Table S2**) from FUSCC. All them were inpatient and had examined the peripheral blood levels of T cell subsets using the flow cytometry at the admission to hospital. Peripheral blood CD8^+^ Tcm was marked by CD3^+^ CD8^+^ CCR7^+^ CD45RA^-^. Peripheral blood CD8^+^ Tem was marked by CD3^+^ CD8^+^ CCR7^-^ CD45RA^-^. Peripheral blood CD8^+^ Tex was marked by CD8^+^ PD1^+^ using the flow cytometry. For patients with LC-BoM, we further divided them into those with low tumor burden of BoM (the number of BoM ≤ 3) and those with high tumor burden of BoM (the number of BoM > 3). The clinical cohort 2 prospectively included 15 patients with newly diagnosed, untreated BoM patients (**Table S3**). The levels of CD8^+^ Tcm, CD8^+^ Tex and CD8^+^ Tem in the peripheral blood of patients before and after DMAb treatment were detected using flow cytometry, and a paired comparison analysis was conducted. The clinical cohort 3 retrospectively comprised patients with spinal BoM from NSCLC who were treated at FUSCC between March 2018 and June 2023. The inclusion criteria were as follows: (1) Patients with BoM accompanied by skeletal-related events (SREs), such as pain or impaired mobility; (2) Pathologically confirmed BoM secondary to NSCLC; (3) Patients who received anti-PD-1 monotherapy, combination anti-angiogenic therapy, or chemotherapy. The types of bone protectants administered to the patients were documented. Patients with incomplete clinical treatment records or survival data were excluded from the analysis. The follow-up period concluded in December 2024.

### 2.15 Transwell assay and in vitro validation

THP-1-derived macrophages were prepared. Briefly, THP-1 cells were seeded into 6-well plates (3×10^5^ cells per well). Phorbol myristate acetate (PMA) was added to a final concentration of 150 nM. The THP-1 cells were incubated in a 37 °C, 5% CO₂ incubator for 24 hours to induce the differentiation of monocytes into macrophages. After 24 hours, the supernatant was removed, and the cells were washed three times with phosphate-buffered saline (PBS) solution. Fresh complete PRMI-1640 medium was added, and the cells were cultured until reaching approximately 80% confluence, followed by collection of M0 macrophages. M1 and M2 macrophages were subsequently induced as appropriate.

We add 500 μL of LC cell suspension at a density of 1×10⁵ cells/mL in complete culture medium to the lower chamber and add 100 μL of macrophage cell suspension to the upper chamber. Cells were incubated at 37°C in a humidified atmosphere containing 5% CO₂ for 24 hours. The surface of the upper chamber membrane was gently wiped with a cotton swab to remove non-invading cells. Cells were fixed with 4% paraformaldehyde at room temperature for 20 minutes. Cells were stained with 0.1% crystal violet for 15 minutes and gently rinsed with PBS. In the intervention experiment, the upper chamber of the CSF1R inhibition group was supplemented with 500 nM CSF1R inhibitor PLX3397, while the upper chamber of the IL6 inhibitor group was supplemented with 50 μmol small molecular interleukin-6 inhibitor LMT-28. Five random fields of view (400× magnification) were selected under an inverted microscope, and the migrated macrophages on the lower surface of the membrane were counted.

Peripheral blood mononuclear cells (PBMCs) were isolated from 8-10 mL of peripheral blood obtained from 7 patients with LC-BoM using the Ficoll-based lymphocyte separation method and subsequently cultured under standard conditions. PBMCs were treated with recombinant human MMP19 protein, while the control group was treated with PBS for 12 hours. Flow cytometry was used to detect the proportion of CD8^+^ T cells. Enzyme-linked immunosorbent assay (ELISA) was used to measure the cytokine of TNF-α secreted by CD8^+^ T cells in the supernatant, which reflect the immune activation status and the antitumor functions of CD8^+^ T cells.

### 2.16 Mouse model for LC-BoM

All animal experiments were conducted in accordance with the protocols approved by the Institutional Animal Care and Use Committee (IACUC) and approved by the ethics committee of FUSCC. LLC-Luc cell lines were maintained and expanded in a complete culture medium supplemented with 1% penicillin-streptomycin and 1 μg/mL puromycin. Female C57BL/6 mice, aged 6 to 8 weeks, purchased from Jihui Biotech. The mice are anesthetized with the isoflurane. After the mouse is fully anesthetized, eye ointment is administered to their eyes to prevent them from drying out. The dorsal skin was disinfected. The position of the ninth thoracic vertebra was identified, and a surgical incision was made to locate the thoracic spinous process and lamina. Subsequently, 50 μL of LLC-Luc cell suspension was injected orthotopically. After the injection was completed, the skin was sutured and the mice were placed. We allowed the mouse to wake up on a heat pad, and reapplied eye ointment if needed. The mouse could be returned to the cage after it showed movement on its own.

The mice that had been successfully modeled were randomly divided into three groups, each containing six mice. The first group received tail vein injections of normal saline and anti-mouse PD-1 (TargetMol, T78269) in 5 mg/kg twice a week; the second group received tail vein injections of zoledronic acid (CSPC) in 100 μg/kg combined with anti-mouse PD-1 (TargetMol, T78269) in 5 mg/kg twice a week; the third group received tail vein injections of RANKL inhibitor OPG protein (TargetMol, TMPJ-00253) in100 μg/kg combined with anti-mouse PD-1 (TargetMol, T78269) in 5 mg/kg twice a week. During the experiment, tumor fluorescence imaging was conducted twice a week, and the lower limb activity function of the mice was observed and recorded. Two weeks later, the mice were euthanized, and the tumor tissue weight and related samples were recorded and collected.

### 2.17 Statistical analysis

Data analysis and graphing were performed using GraphPad Prism version 9.5. Continuous data were presented as mean ± standard deviation (SD) or median with quartiles. Categorical data were presented as number with frequency. Student's t-test or the nonparametric Kruskal-Wallis test was used to compare two independent groups, as appropriate. One-way ANOVA was used to compare differences among multiple groups. Survival curves were constructed using the Kaplan-Meier method and compared by means of the log-rank test. The multivariate Cox proportional hazards regression model was employed to identify independent prognostic factors and to estimate hazard ratios (HR) with 95% confidence intervals (CI). A P value of less than 0.05 was considered statistically significant (*P < 0.05; **P < 0.01; and ***P < 0.001).

## 3. Results

### 3.1 Cellular constitution between primary tumors and BoM of PC, LC, and BC, respectively

**Figure 1:**
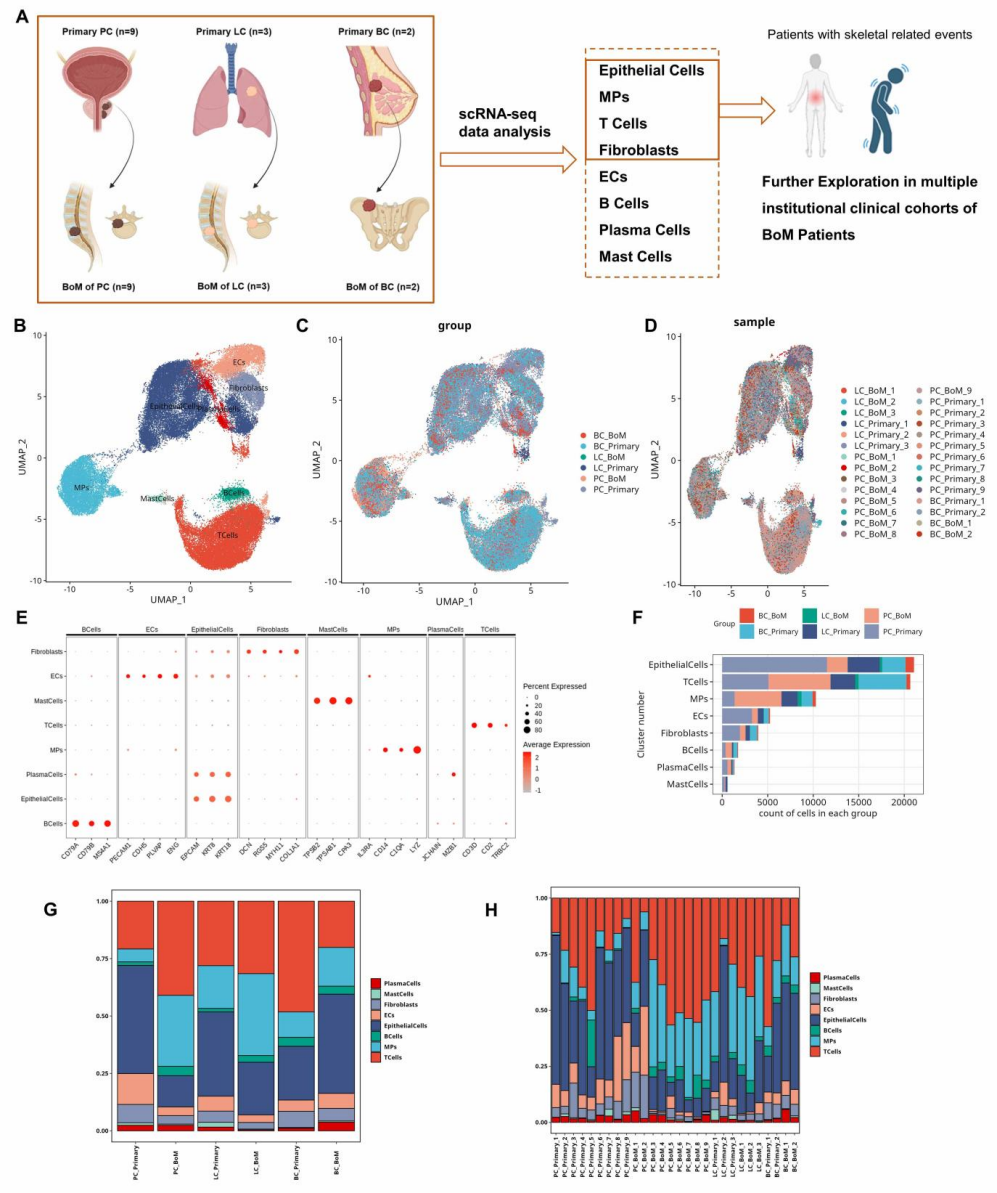
** Cellular constitutions in primary tumor and bone metastasis (BoM) of prostate cancer (PC), lung cancer (LC), and breast cancer (BC) at single-cell resolution. A:** Study workflow chart. **B:** UMAP plot illustrating eight major cell types (n= 6,4940 cells).** C:** UMAP plot illustrating origin of these cells from specific cancer types/groups (n= 6 groups). **D:** UMAP plot illustrating origin of these cells from each sample (n= 28 samples). **E:** Dot plot illustrating canonical cell marker expression of the eight major cell types. **F:** Bar chart illustrating the number of cells. Epithelial, T and MPs cells constitute the majority of the tumor ecosystem. **G and H:** The proportion of major cell subsets in each group (G) and sample (H) showed that MPs is commonly increased in BoM compared to primary tumor.

To comprehensively understand the role of tumor microenvironment in BoM of PC, LC, and BC, we retrieved scRNA-seq data from 28 tumor samples (**Table S1**), including (1) PC group (n=18): 9 PC primary tumors (PC_Primary) and 9 PC bone metastasis (PC_BoM) tissues; (2) LC group (n=6): 3 NSCLC primary tumors (LC_Primary) and 3 NSCLC bone metastasis (LC_BoM) tissues; (3) BC group (n=4): 2 BC primary tumors (BC_Primary) and 2 BC bone metastasis (BC_BoM) tissues (**Figure 1A**). All scRNA-seq data were sequenced via 10× Genomics platform.

After cell quality control with double-cell filtration (**Figure S1A and B**), a total of 6,4940 cells were obtained and forwarded into the subsequent analysis. Based on the canonical cell markers, we divided these 6,4940 cells into eight subsets on a uniform manifold approximation and projection (UMAP) plot, including epithelial cells, T cells, myeloid cells/mononuclear macrophages (MPs), endothelial cells (ECs), fibroblasts, B cells, mast cells, and plasma cells (**Figure 1B**). The origin of these cells from specific tumor types/groups (**Figure 1C**) and samples (**Figure 1D**) were displayed. Epithelial cell markers were EPCAM, KRT8, and KRT18. MPs cell markers were IL3RA, CD14, C1QA and LYZ. T cell markers were CD3D, CD2 and TRBC2. B cell markers were CD79A, CD79B, and MS4A1. ECs markers were PECAM1, CDH5, PLVAP, and ENG. Fibroblast markers were DCN, RGS5, MYH11 and COL1A1. Mast cell markers were TPSB2, TPSAB1 and CPA3. Plasma cell markers were CD79A, JCHAIN and MZB1 (**Figure 1E**). Top ten highly expressed DEGs of each cell subsets were identified (**Figure S1C**).

In the tumor ecosystem, epithelial cells (tumor cells) accounted for the largest number, followed by T cells, and myeloid cells/MPs (**Figure 1F**). Therefore, in this study, we would focus on exploring the epithelial/tumor, T and myeloid/MPs cells and their heterogeneity in BoM. For PC, LC, and BC, the proportion of myeloid cells was consistently increased in BoM compared to primary tumor (**Figure 1G and H**).

### 3.2 Tumor cells heterogeneity between primary tumors and BoM across PC, LC, and BC

**Figure 2:**
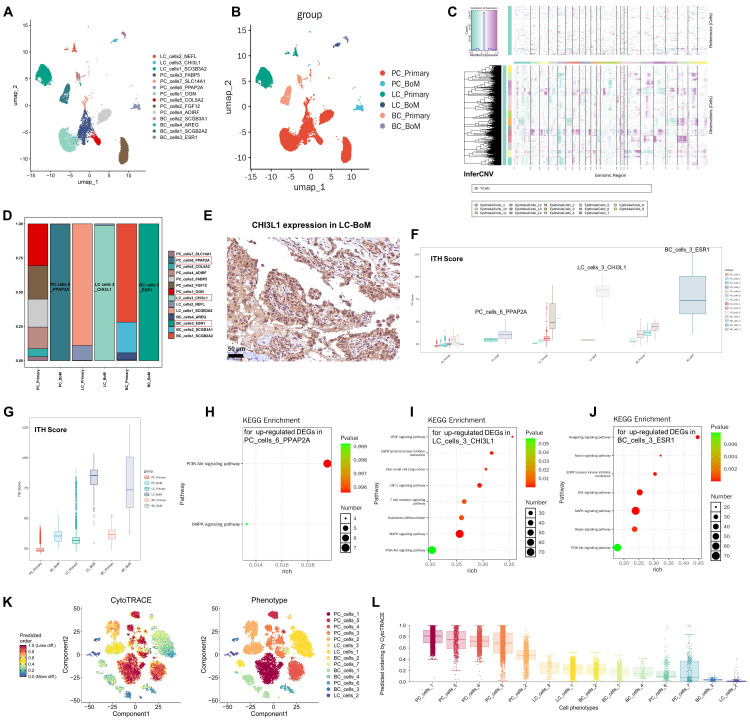
** Tumor cells heterogeneity between primary tumor and BoM across prostate cancer (PC), lung cancer (LC), and breast cancer (BC). A:** UMAP plot illustrating 7 PC cells clusters (PC_cells_1~7), 3 LC cells clusters (LC_cells_1~3) and 4 BC cells clusters (BC_cells_1~4) with their cell maker. **B:** UMAP plot illustrating the origin of the tumor cell subclusters from specific tumor types/groups. **C:** Copy number variation (CNV) heatmap shows that the fourteen tumor subclusters present increased CNV levels compared to T cells. The X-axis represents the chromosome, the Y-axis represents the epithelial subclusters. **D:** Bar chart illustrating the proportion of cancer cell subsets between primary tumor and BoM. **E:** The expression pattern of CHI3L1 in LC-BoM using the immumohistochemical (IHC) staining. CHI3L1 was strongly expressed in the cytoplasm of LAUD cells and diffusely spread in the tumor stroma of BoM. Scale = 50 μm. **F and G:** Intra-tumor heterogeneity (ITH) score for different tumor cell subclusters and tumor types. Of tumor cells, LC_cells_3_CHI3L1 displays the highest ITH score. Of tumor types, LC-BoM displays the highest ITH. The higher ITH, the worse effect of immunotherapy for cancer. **H, I, and J:** KEGG pathway enrichment analysis for the up-regulated DEGs in PC_cells_6_PPAP2A, LC_cells_3_CHI3L1 and BC_cells_3_ESR1, respectively. **K and L**: Cell stemness evaluation by CytoTRACE. CytoTRACE values range from 0 (low stemness, highly differentiated) to 1 (high stemness, less differentiated).

Epithelial cells were divided into fourteen subclusters based on the UMAP (**Figure S2A**). Mapping the 14 subsets on the sample types origin, we identify 7 PC cells clusters (PC_cells_1~7), 3 LC cells clusters (LC_cells_1~3) and 4 BC cells clusters (BC_cells_1~4) (**Figure S2B; Figure 2A**). The origin of the fourteen subclusters from specific tumor types/groups were displayed (**Figure 2B**). We used copy number variation (CNV) analysis to identify malignant tumor cells. All the 14 epithelial subclusters had elevated CNV levels compared to T cells and thus were considered as malignant tumor cells (**Figure 2C; Figure S2 C**).

Compared to primary PC, cancer cells of PC-BoM were nearly all PC_cells_6 which was characterized by phosphatidic acid phosphatase type 2A (PPAP2A) expression (**Figure 2D**). PPAP2A has been reported as a new cancer gene of PC 15. The external data in The Cancer Genome Atlas (TCGA) available within GEPIA2 validated that PPAP2A expression increased in PC tissues compared to the normal prostate tissues (**Figure S2D**). PPAP2A^+^ PC cells might be a key driver of PC-BoM.

Compared to primary LC, cancer cells of LC-BoM were nearly all LC_cells_3 which was characterized by chitinase-3-like 1 (CHI3L1) expression (**Figure 2D**). Immumohistochemical (IHC) staining verified that CHI3L1 was strongly expressed in the cytoplasm of LUAD cells and diffusely secreted into tumor stroma of LC-BoM tissue (**Figure 2E**). CHI3L1 was moderately expressed in the cytoplasm of LUAD cells, but barely in tumor stroma of primary LC tissue (**Figure S2E**).

A latest study has reported that CHI3L1 is involved in the pathogenesis of epidermal growth factor receptor (EGFR)-mutant NSCLC 16. CHI3L1 expression was increased in LUAD and LUSC tissues compared to the normal lung tissues (**Figure S2F**). CHI3L1 is reported as a marker for circulating tumor cells of LC and associated with cancer cell dissemination in the peripheral blood 17. Inhibition of CHI3L1 could suppress LC growth and metastasis 18,19. Our result further suggested that CHI3L1^+^ LC cells might be the most key driver of LC-BoM.

Compared to primary BC, cancer cells of BC-BoM were nearly all BC_cells_3 which was characterized by estrogen receptor 1 (ESR1) expression (**Figure 2D**). ESR1 expression was increased in BC tissues compared to the normal breast tissues (**Figure S2G**). ESR1 mutation is associated with high estrogen receptor expression. Its well-known that BC patients with positive estrogen receptor are more likely to undergo BoM 20, 21, 22. Being consistent with those studies, we found ESR1^+^ BC cells might be associated with BC-BoM.

Intra-tumor heterogeneity (ITH) reflects the difference in genetic expression profiles between different cells within a solid tumor. The higher ITH, the more complex interactions between tumor cells and the tumor microenvironment. High ITH drives tumor cell evolution, treatment resistance and recurrence. Generally, the higher ITH, the worse effect of immunotherapy for cancer 23. In our study, all BoM demonstrated markedly higher ITH score than primary tumors in PC, LC and BC groups (**Figure 2F and G**). LC_cells_3_CHI3L1 presented the highest ITH among all tumor subsets. LC_cells_3_CHI3L1 might be a strong initiator of immunotherapy resistance in LC-BoM.

The up-regulated DEGs were enriched via KEGG (Kyoto Encyclopedia of Genes and Genomes) pathway analysis for PC_cells_6_PPAP2A (**Figure 2H**), LC_cells_3_CHI3L1 (**Figure 2I**) and BC_cells_3_ESR1 (**Figure 2J**). In addition, LC_cells_3_CHI3L1 demonstrated up-regulated signaling pathways of vascular endothelial growth factor (VEGF), EGFR tyrosine kinase inhibitor (TKI) resistance and osteoclast differentiation.

Cell stemness evaluated by the CytoTRACE analysis demonstrated that PC_cells_6_PPAP2A and BC_cells_3_ESR1 had low stemness scores and were highly differentiated. LC_cells_3_CHI3L1 had high stemness score and was less differentiated compared to LC_cells_1 and LC_cells_2 (**Figure 2K and L**).

### 3.3 MPs heterogeneity between primary tumors and BoM across PC, LC, and BC

**Figure 3:**
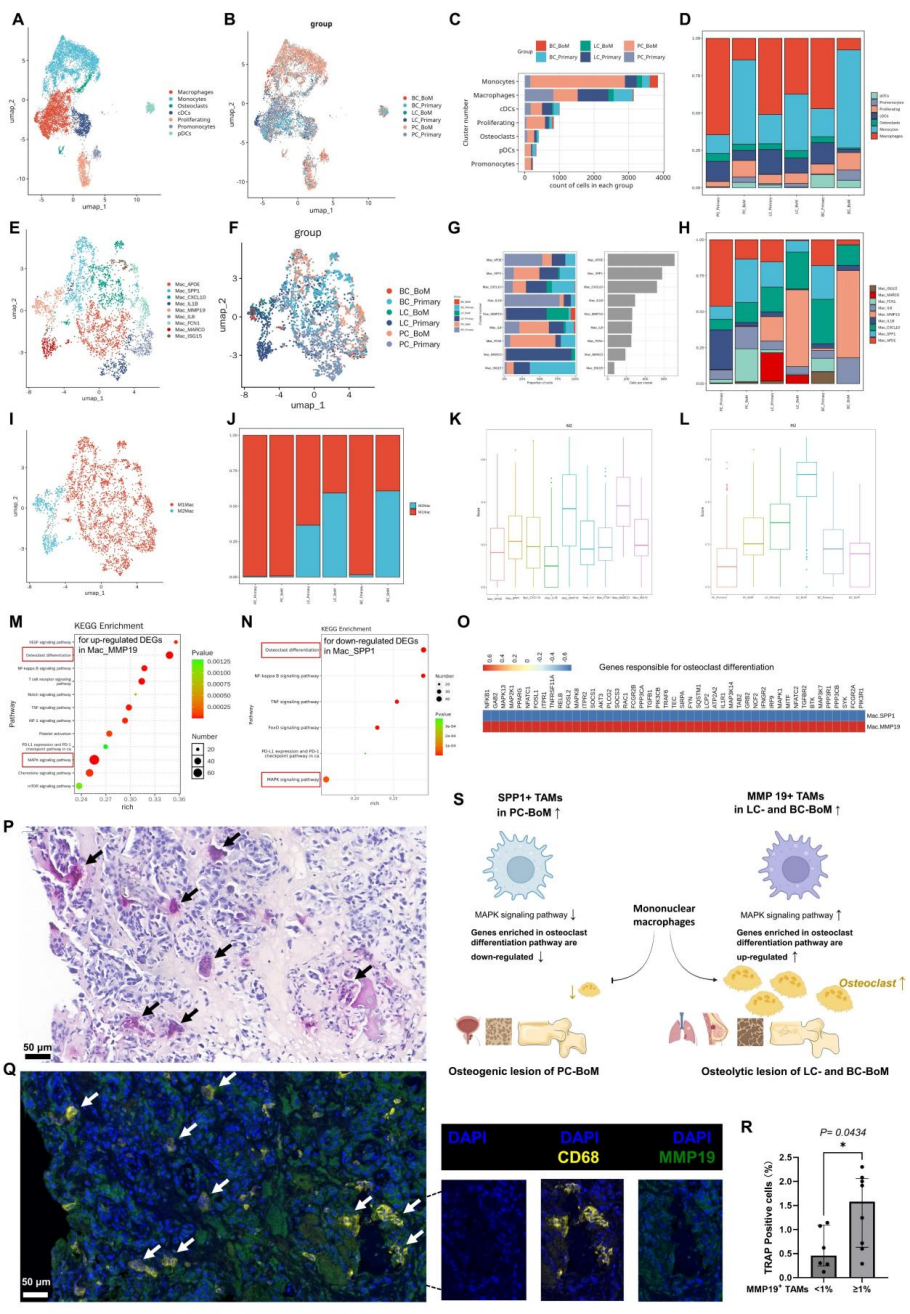
** Myeloid cells heterogeneity between primary lesion and BoM of prostate cancer (PC), lung cancer (LC), and breast cancer (BC). A:** UMAP plot illustrating the seven subsets of myeloid cells. **B:** UMAP plot illustrating the origin of the myeloid cells subsets from specific tumor types/groups. **C:** Bar chart illustrating the number of the 7 myeloid cells subsets. Monocytes have the largest number, followed by tumor-associated macrophages (TAMs). **D**: Bar chart illustrating the proportion of different myeloid cells subsets between primary lesion and BoM. The proportion of TAMs consistently decreased in BoM compared to primary lesions for PC, LC and BC. **E**: UMAP plot illustrating the 9 subsets of TAMs. **F:** UMAP plot illustrating the origin of the TAMs subsets from specific tumor types/groups. **G:** Bar chart illustrating the number of the 9 subsets of TAMs. **H**: Bar chart illustrating the proportion of different TAMs subsets between primary lesion and BoM. The proportion of Mac_SPP1 increased in PC-BoM compared to primary PC. The proportion of Mac_MMP19 consistently increased in LC-BoM and BC-BoM compared to primary LC and BC lesions, respectively. **I:** UMAP plot illustrating the M1 and M2 typing of TAMs. **J:** Bar chart illustrating the proportion of M2-TAMs between primary lesions and BoM. The proportion of M2-TAMs consistently increased in LC-BoM and BC-BoM compared to the corresponding primary lesions. **K:** M2-macrophages score in the 9 subsets of TAMs. Mac_MMP19 and Mac_MACRO exhibit the highest M2 score. **L:** M2-macrophages score in the 6 specific tumor types/groups. LC-BoM exhibits the highest M2 score. **M:** KEGG (Kyoto Encyclopedia of Genes and Genomes) pathway enrichment analysis for the up-regulated differentially expressed genes (DEGs) in Mac_MMP19. The up-regulated DEGs of Mac_MMP19 were enriched in osteoclast differentiation and MAPK signal pathways. **N**: KEGG pathway enrichment analysis for the down-regulated DEGs in Mac_SPP1. The down-regulated DEGs of Mac_SPP1 were enriched in osteoclast differentiation and MAPK signal pathways. **O**: The expression level of key genes responsible for osteoclast differentiation in Mac_SPP1 and Mac_MMP19. **P**: Tartrate-resistant acid phosphase (TRAP) staining showed that a large number of osteoclasts exist in the LC-BoM tissues that have not been clinically treated. Continuous sections of the same slice as Figure 3Q. **Q**: Immunofluorescence staining showed that Mac_MMP19 marked by the co-expression of CD68 and MMP19, exhibited a co-localization and/or closely adjacent localization with osteoclasts. Continuous sections of the same slice as Figure 3P. **R**: Quantitative analysis of Figure P and Q revealed that regions with high Mac_MMP19 distribution also had a greater number of osteoclasts. **S**: Schematic diagram elucidating that LC-BoM and BC-BoM are more osteolytic but PC-BoM is less osteolytic from the view of TAMs heterogeneity.

Myeloid cells/MPs were divided into macrophages, monocytes, osteoclasts, conventional dendritic cells (cDCs), proliferating myeloid cells, promoctyes, and plasmacytoid dendritic cells (pDCs) based on the UMAP (**Figure 3A**). The origin and cell number of the myeloid cells subsets from specific tumor types/groups were displayed (**Figure 3B and C**). Marker genes expression of the myeloid cells subsets was displayed (**Figure S3 A**). The proportion of tumor-associated macrophages (TAMs) consistently decreased in PC-BoM, LC-BoM, and BC-BoM compared to their corresponding primary lesions (**Figure 3D**).

We further subdivided TAMs into 9 subtypes, including Mac_APOE, Mac_SPP1, Mac_CXCL10, Mac_IL1B, Mac_MMP19, Mac_IL8, Mac_FCN1, Mac_MARCO, and Mac_ISG15 (**Figure 3E**). The origin and cell number of the TAMs subtypes from specific tumor types/groups were displayed (**Figure 3F and G**).

The proportion of matrix metalloproteinase 19 (MMP19)^+^ macrophages (Mac_MMP19) consistently increased in LC-BoM and BC-BoM compared to primary LC and BC lesions, respectively (**Figure 3H**); Mac_MMP19 may serve as a common driver both for LC-BoM and BC-BoM.

Subdivision of TAMs into M1 and M2 subtypes was also performed (**Figure 3I**) based on the canonical marker gene expression (**Figure S3B**). The proportion of M2-TAMs consistently increased in LC-BoM and BC-BoM compared to the primary LC and BC lesions, respectively (**Figure 3J**). M2-macrophages score was highest in Mac_MMP19 (**Figure 3K**); and in LC-BoM (**Figure 3L**), respectively. Immunofluorescence staining further verified that M2-TAMs were widely expressed in LC-BoM tissues (**Figure S3C**). Those results suggested M2 polarization plays an important role driving LC-BoM.

Mac_MMP19 had up-regulated osteoclast differentiation and MAPK signal pathways (**Figure 3M**). By contrast, Mac_SPP1 had down-regulated osteoclast differentiation and MAPK signal pathways (**Figure 3N**). Key genes responsible for osteoclast differentiation were down-regulated in Mac_SPP1, but up-regulated in Mac_MMP19 (**Figure 3O**).

Tartrate-resistant acid phosphase (TRAP) staining showed that osteoclasts were abound in the mesenchyme of treatment-naive LC-BoM (**Figure 3P**). Multi-immunofluorescence (mIF) staining further demonstrated that Mac_MMP19, identified by the co-expression of CD68 and MMP19, exhibited co-localization or close proximity to osteoclasts (**Figure 3Q**). The areas with higher proportion of MMP19^+^ TAMs contain higher proportion of osteoclasts (TRAP^+^) (**Figure 3R**). Those results suggested that MMP19^+^ TAMs represented a precursor of osteoclasts and contributed to osteoclast information in LC-BoM. This findings provide insight into the underlying reasons why LC-BoM and BC-BoM exhibit a higher degree of osteolysis, while PC-BoM exhibits a lower degree of osteolysis in terms of TAM heterogeneity (**Figure 3S**).

### 3.4 Tumor necrosis factor receptor superfamily-member 11A (TNFRSF11A)/ receptor activator of nuclear factor-κB (RANK) expression on TAMs and the effect of DMAb in LC-BoM

TNFRSF11A is the encoding gene of RANK. TNFRSF11A is prominently expressed in osteoclasts and encodes the expression of RANK on osteoclasts. Receptor Activator for Nuclear factor-κB ligand (RANKL) is the specific ligand of RANK. RANKL promotes osteoclast differentiation, maturation, and functional activity through its binding to RANK 24,25. Osteoprotegerin (OPG) acts as a soluble decoy receptor for RANKL, thereby preventing RANKL from binding to its receptor, RANK, and ultimately inhibiting the process of osteoclastogenesis 26,27. Denosumab (DMAb) is a humanized monoclonal antibody that exhibits functional similarities to osteoprotegerin (OPG). DMAb is able to bind competitively to the human RANKL protein, thereby inhibiting the interaction between RANKL and RANK on the surface of osteoclasts. Consequently, DMAb effectively suppresses the differentiation and activation of osteoclasts, ultimately leading to a reduction in bone resorption and lysis 28-30.

**Figure 4:**
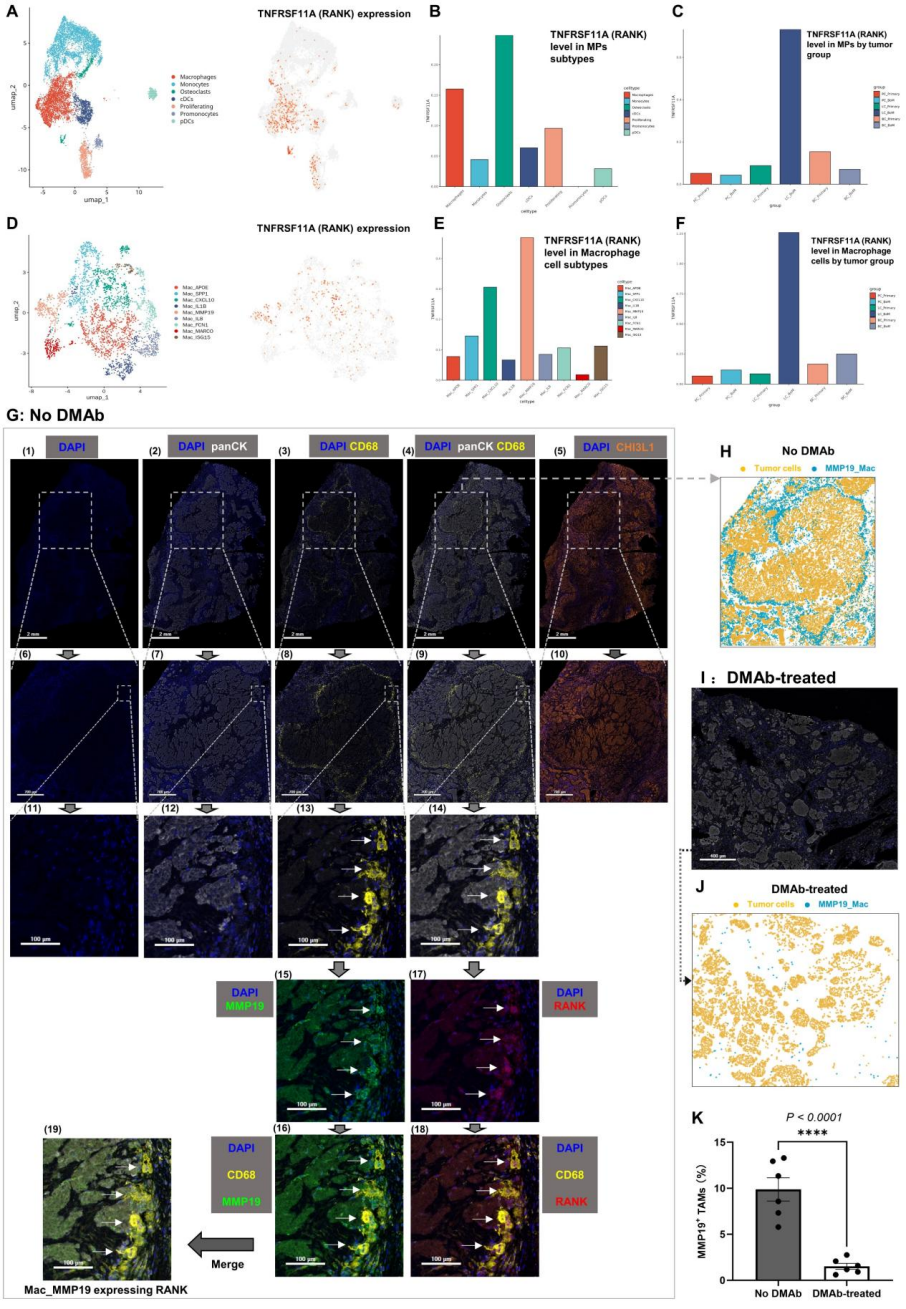
** Tumor necrosis factor receptor superfamily-member 11A (TNFRSF11A)/ receptor activator of nuclear factor-κB (RANK) expression on tumor-associated macrophages (TAMs). A:** UMAP plot illustrating the expression of TNFRSF11A/RANK in seven subsets of myeloid cells. **B**: Bar chart illustrating the expression levels of TNFRSF11A/RANK in seven subsets of myeloid cells. Osteoclasts and macrophages exhibit remarkably high expression levels of TNFRSF11A/RANK. **C:** Bar chart illustrating the expression levels of TNFRSF11A/RANK in myeloid cells across six tumor groups. Myeloid cells in LC-BoM exhibited the highest expression levels of TNFRSF11A/RANK. **D**: UMAP plot illustrating the expression of TNFRSF11A/RANK in nine subsets of TAMs. **E**: Bar chart illustrating the expression levels of TNFRSF11A/RANK in nine subsets of TAMs. Mac_MMP19 exhibited the highest expression levels of TNFRSF11A/RANK. **F**: Bar chart illustrating the expression levels of TNFRSF11A/RANK in TAMs across six tumor groups. TAMs in LC-BoM exhibited the highest expression levels of TNFRSF11A/RANK. **G**: Multi-immunofluorescence (mIF) staining for LC-BoM tissues with no DMAb-treatment. DAPI is used to stain cell nuclei, panCK is utilized as a marker for cancer cells, and CD68 is utilized as a marker for TAMs. TAMs were arranged in a ring-like pattern surrounding the bone metastatic LC cells nests (Figure 4G 4). The bone metastatic LC cells highly expressed CHI3L1 (Figure 4G 5). TAMs characterized by CD68 expression in LC-BoM demonstrated high co-expression levels of MMP19 and RANK (Figure 4G 14-19). **H**: A pattern diagram was generated by the HALO software based on the Figure 4G (9). MMP19^+^ TAMs form a ring-like structure surrounding the bone metastatic LC cells. **I**: mIF staining for LC-BoM tissues with DMAb-treatment. **J**: A pattern diagram was generated by the HALO software based on the Figure 4I. The bone metastatic LC cells were not surrounded by MMP19^+^ TAMs. **K**: Quantitative analysis by the HALO software demonstrated that the density of MMP19^+^ TAMs in DMAb-treated LC-BoM tissues (N=6) was significantly lower compared to that in LC-BoM tissues without DMAb (N=6). MMP19^+^ TAMs (%) refers to the proportion of this type of cells among the total cell count.

In our study, osteoclasts demonstrated the highest level of TNFRSF11A (RANK) expression among all myeloid cell types examined. Furthermore, our analysis revealed that macrophages (TAMs) exhibit high expression levels of TNFRSF11A (RANK), with its expression level ranking second only to that observed in osteoclasts (**Figures 4A and B**).

The expression level of TNFRSF11A (RANK) in MPs cells was highest in LC-BoM compared to other tumor tissues (**Figure 4C**). Among the nine TAMs subtypes, Mac_MMP19 demonstrated the highest expression level of TNFRSF11A (RANK) (**Figure 4D and E**). The expression level of TNFRSF11A (RANK) in TAMs was highest in LC-BoM compared to other tumor tissues (**Figure 4F**).

The mIF staining was conducted to validate the above scRNA-seq findings in treatment-naïve LC-BoM samples (samples of LC-BoM that had not been treated with DMAb). In the LC-BoM with no DMAb treatment, the TAMs that exhibited high co-expression of MMP19 and RANK were arranged in a ring-like pattern surrounding the nests of bone metastatic LC cells with high expression of CHI3L1 (**Figure 4G**). A pattern diagram generated by the HALO software illustrated the phenomenon of MMP19^+^ TAMs surrounding the bone metastatic LC cells (**Figure 4H**). By contrast, MMP19^+^ TAMs were scarcely detected in LC-BoM samples treated with DMAb (**Figure 4I**). The model diagram indicated the absence of the phenomenon where MMP19^+^ TAMs form a ring around tumor cells in DMAb-treated LC-BoM samples (**Figure 4J**). Quantitative analysis demonstrated that DMAb treatment was significantly associated with reduced density of MMP19^+^ TAMs (**Figure 4K**).

### 3.5 T cells and fibroblasts heterogeneity between primary tumors and BoM across PC, LC and BC

**Figure 5:**
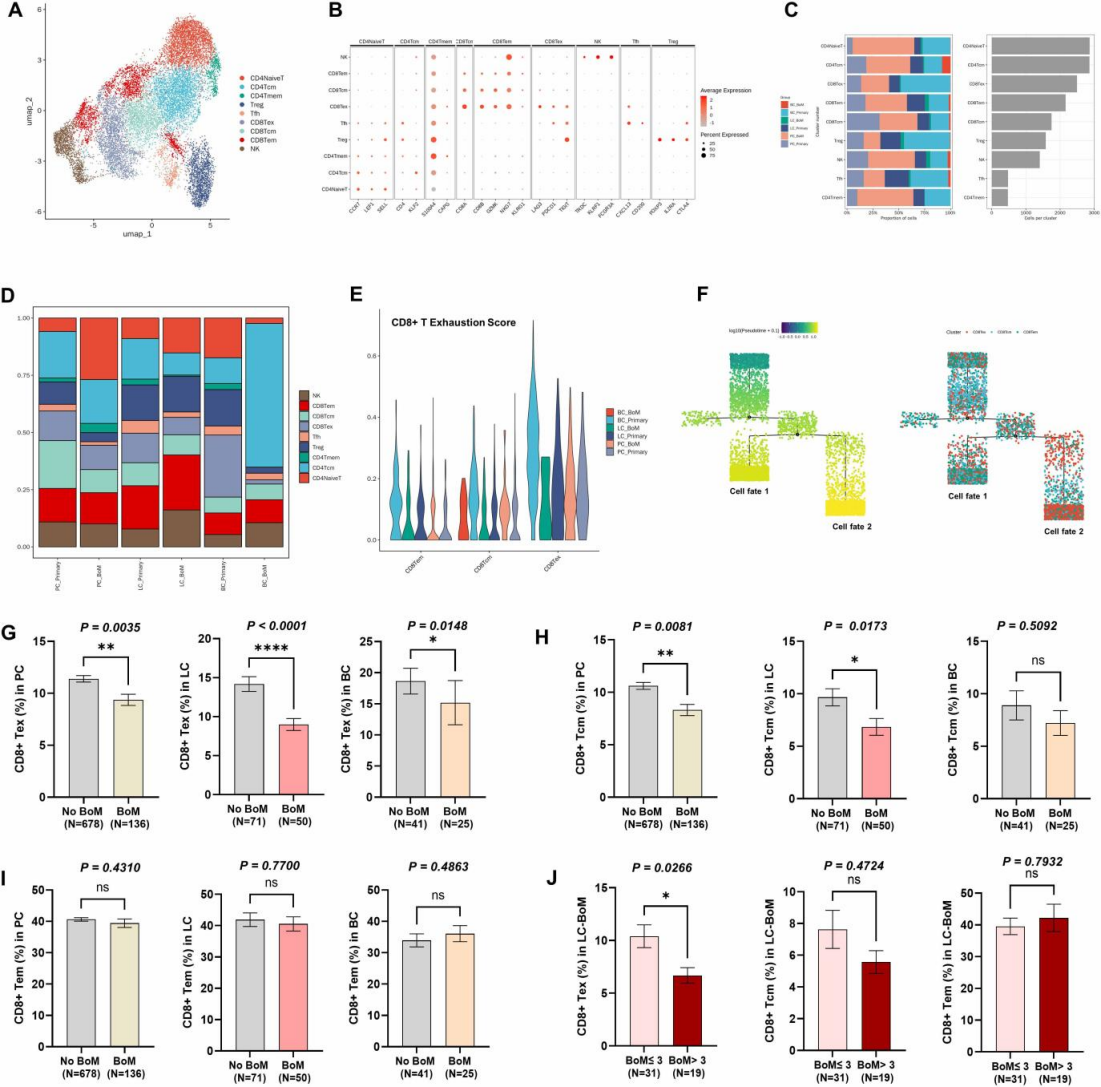
** T cells heterogeneity between primary tumor and BoM of prostate cancer (PC), lung cancer (LC), and breast cancer (BC), respectively. A**: UMAP plot illustrating the nine subsets of T cells. **B**: Dot plot illustrating canonical cell marker expression of the nine subsets of T cells. **C**: Bar chart illustrating the proportion and number of the nine subsets of T cells. **D**: Bar chart illustrating the proportion of the T cell subsets between primary lesion and BoM. The proportion of CD8^+^ Tex was consistently observed to decrease in BoM compared to primary lesions across PC, LC and BC. The proportion of CD8^+^ Tcm was consistently observed to decrease in BoM compared to primary lesions in PC and LC, but no significant change was observed in BC. **E**: The T-cell exhaustion score analysis revealed that LC-BoM had a lower score compared to the primary lesion. **F**: The pseudo-time tree plot revealed the CD8^+^ Tex at the terminal stage of CD8^+^ T cell differentiation. **G**: Clinical cohort 1 revealed the level of CD8^+^ Tex in PC-BoM, LC-BoM, and BC-BoM groups. **H**: Clinical cohort 1 revealed the level of CD8^+^ Tcm in PC-BoM, LC-BoM, and BC-BoM groups. **I**: Clinical cohort 1 revealed the level of CD8^+^ Tem in PC-BoM, LC-BoM, and BC-BoM groups. **J**: Clinical cohort 1 revealed the level of CD8^+^ Tem, Tcm and Tem between high and low BoM groups within LC-BoM patients. Of 50 patients with LC-BoM, those with high burden of BoM (the number of BoM > 3; n=19) had significantly lower peripheral CD8^+^ Tex level compared to those with low burden of BoM (the number of BoM ≤ 3; n=31). The higher burden of LC-BoM, the lower peripheral levels of CD8^+^ Tex.

T cells were divided into nine subsets based on the UMAP (**Figure 5A**), including CD4^+^ naïve T, CD4^+^ central memory T (CD4^+^ Tcm), CD4^+^ memory T (CD4^+^ Tmem), CD4^+^ regulatory T (Treg), follicular helper T (Tfh), CD8^+^ exhausted T (CD8^+^ Tex), CD8^+^ central memory T (CD8^+^ Tcm), CD8^+^ effector memory T (CD8^+^ Tem) and nature killer (NK) cells. Marker genes expression (**Figure 5B**) of each T cell subset was depicted. CD4^+^ naïve T and CD4^+^ Tcm accounted for the highest number, followed by CD8^+^ Tex, CD8^+^ Tem, and CD8^+^ Tcm (**Figure 5C**).

In intergroup comparisons, the proportion of CD8^+^ Tex was consistently observed to decrease in the PC-BoM, LC-BoM, and BC-BoM groups when compared to their corresponding primary lesions. The proportion of CD8^+^ Tcm was found to consistently decrease in both the PC-BoM and LC-BoM groups when compared to their corresponding primary lesions, whereas no significant change was observed in the BC-BoM group. The proportion of CD8^+^ Tem in the PC-BoM, LC-BoM, and BC-BoM groups was comparable to that in their corresponding primary lesions (**Figure 5D**). Compared to the primary lesion of LC, the T-cell exhaustion score in LC-BoM was lowered (**Figure 5E**).

Pseudo-temporal trajectory analysis demonstrated that CD8^+^ Tcm and CD8^+^ Tem cells were predominantly positioned at the initiation of the cell differentiation trajectory, whereas CD8^+^ Tex cells were primarily localized at the terminal end of this trajectory (**Figure 5F**).

To validate the findings derived from scRNA-seq, we retrospectively enrolled a substantial cohort of patients with PC, LC, and BC, both with and without BoM, from Fudan University Shanghai Cancer Center (FUSCC) (**Clinical cohort 1 at FUSCC**, total n=1,001). The clinical cohort 1 included 814 PC patient, of whom 136 had BoM and 678 had no BoM; 121 LC patients, of whom 50 had BoM and 71 had no BoM; 66 BC patients, of whom 25 had BoM and 41 had no BoM (**Supplementary Table S2**). All the patients underwent examination of their peripheral blood levels for CD8^+^ Tex, CD8^+^ Tcm, and CD8^+^ Tem using flow cytometry.

The level of CD8^+^ Tex was significantly decreased in patients with BoM compared to those without BoM across PC, LC, and BC groups (**Figure 5G**). Patients with BoM exhibited significantly reduced levels of CD8^+^ Tcm compared to those without BoM, both in the PC and LC groups, but no significant difference was observed in the BC group (**Figure 5H**). Patients with BoM exhibited comparable levels of CD8+ Tem cells relative to patients without BoM in PC, LC, and BC (**Figure 5I**).

Among the 50 patients with LC-BoM, 31 patients presented with no more than three BoM lesions and were categorized as having a low BoM burden. Conversely, 19 patients presented with more than three BoM lesions and were categorized as having a high BoM burden. Notably, a high BoM burden (BoM > 3 lesions) was associated with significantly lower levels of CD8^+^ Tex, while no significant association was observed with CD8^+^ Tcm or CD8^+^ Tem (**Figure 5J**).

### 3.6 The prognostic significance of CD8^+^ T cell subsets across PC, LC and BC

The prognostic significance of CD8^+^ T cell subsets was investigated by leveraging the TCGA/GTEx datasets in GEPIA2 and the follow-up data from the Clinical Cohort 1 at FUSCC, respectively.

**Figure 6:**
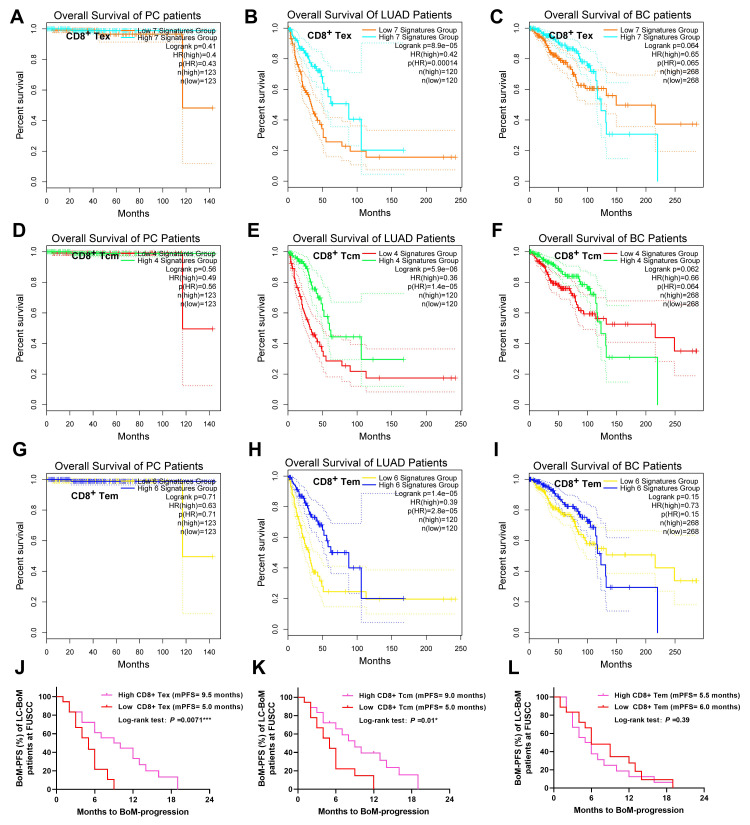
** The prognostic significance of CD8^+^ T cell subsets.** CD8^+^ Tex-associated gene signatures comprised seven genes: HAVCR2, TIGIT, LAG3, PDCD1, CXCL13, LAYN, and CD8. CD8^+^ Tcm-associated gene signatures comprised four genes: CCR7, SELL, IL7R, and CD8. CD8^+^ Tem-associated gene signatures comprised six genes: PDCD1, DUSP4, GZMK, GZMA, IFNG, and CD8. The classification of high or low is determined based on the quartiles of the signature expression level. CD8^+^ Tcm is associated with T cell rapid proliferation and differentiation, long-term immune memory and lymphoid homing and distribution. CD8^+^ Tem is associated with rapid killing of tumor cells and secretion of cytokines. CD8+Tex can reverse its exhausted state and restore its anti-tumor function through immune checkpoint blockade. **A**: No difference in OS among PC patients stratified by CD8^+^ Tex gene signatures. **B:** Significant difference in OS among LUAD patients stratified by CD8^+^ Tex gene signatures. **C:** No difference in OS among BC patients stratified by CD8^+^ Tex gene signatures. **D**: No significant difference in OS among PC patients stratified by CD8^+^ Tcm gene signatures. **E:** Significant difference in OS among LUAD patients stratified by CD8^+^ Tcm gene signatures. **F:** No significant difference in OS among BC patients stratified by CD8^+^ Tcm gene signatures. **G**: No significant difference in OS among PC patients stratified by CD8^+^ Tem gene signatures. **H:** Significant difference in OS among LUAD patients stratified by CD8^+^ Tem gene signatures. **I:** No significant difference in OS among BC patients stratified by CD8^+^ Tem gene signatures. **J:** Low level of peripheral blood CD8^+^ Tex was associated with shorter median BoM-progression-free survival (BoM-PFS) in LC-BoM patients. **K**: Low level of peripheral blood CD8^+^ Tcm was associated with shorter median BoM-PFS in LC-BoM patients. **L**: Peripheral blood CD8^+^ Tem was not associated with BoM-PFS in LC-BoM patients.

Low expression levels of CD8^+^ Tex gene signatures were significantly associated with poorer OS in LUAD tissues, whereas no significant association was observed in PC and BC tissues (**Figure 6A, B and C**). Low expression levels of CD8^+^ Tcm gene signatures were significantly associated with poorer OS in LUAD tissues, whereas no significant association was observed in PC and BC tissues (**Figure 6D, E, and F**). Low expression levels of CD8^+^ Tem gene signatures were significantly associated with poorer OS in LUAD tissues, whereas no significant association was observed in PC and BC tissues (**Figure 6G, H, and I**).

Based on the above results, using the Clinical cohort 1 at FUSCC, we further validated the prognostic significance of peripheral blood CD8^+^ T cell subsets in patients with LC-BoM treated with anti-PD1 immunotherapy. Among the 50 LC-BoM patients at FUSCC, 36 received subsequent systemic therapy containing anti-PD1 immunotherapy. Low levels of peripheral blood CD8^+^ Tex and Tcm, but not Tem, were associated with shorter median BoM-progression-free survival (BoM-PFS) (**Figure 6J, K, and L**).

### 3.7 The impact of DMAb on T cell subsets in LC-BoM and its clinical efficacy when combined with anti-PD1 immunotherapy

**Figure 7:**
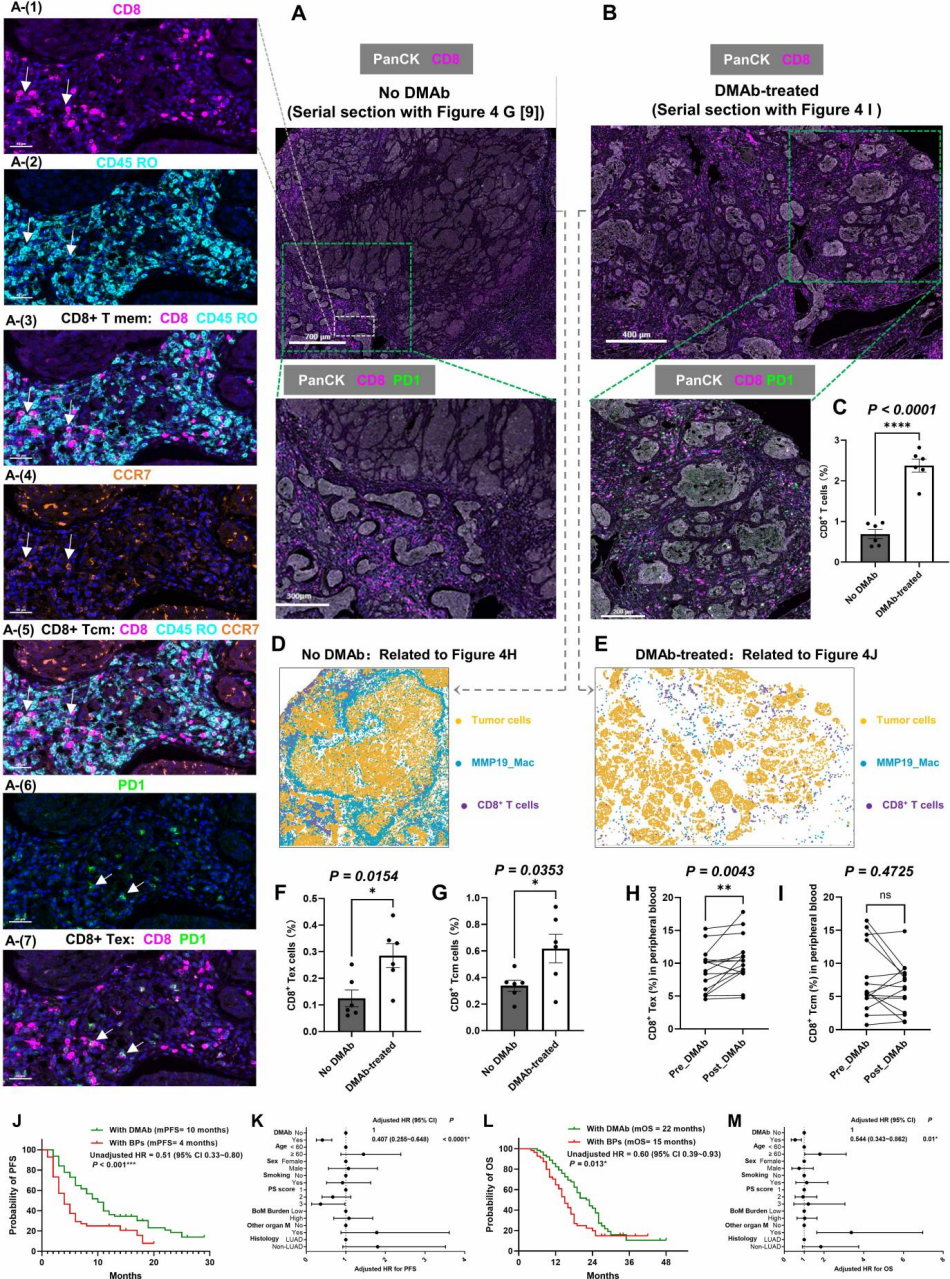
** The impact of Denosumab (DMAb) on T cell subsets in LC-BoM and its clinical efficacy when combined with anti-PD1 immunotherapy. A**: mIF staining for T cell subsets in the LC-BoM tissues not treated with DMAb (serial section with Figure 4G 9). **B**: mIF staining for T cell subsets in the LC-BoM tissues treated with DMAb (serial section with Figure 4I). **C**: The LC-BoM tissues treated with DMAb exhibited significantly greater CD8^+^ T cell infiltration compared to those not treated with DMAb. **D**: A pattern diagram was generated by the HALO software based on the Figure 7A. In untreated LC-BoM tissues, MMP19^+^ TAMs form a circular barrier that inhibits T cell infiltration into the inner region of the tumor. **E**: A pattern diagram was generated by the HALO software based on the Figure 7B. In DMAb-treated LC-BoM tissues, the ring-like barrier of MMP19^+^ TAMs was eliminated, CD8^+^ T cells were massively infiltrated around the tumor nest. **F**: Quantitative analysis using the HALO software revealed a significantly higher density of CD8^+^ Tex in DMAb-treated LC-BoM tissues (N=6) compared to untreated LC-BoM tissues (N=6). **G**: Quantitative analysis using the HALO software revealed a significantly higher density of CD8^+^ Tcm in DMAb-treated LC-BoM tissues (N=6) compared to untreated LC-BoM tissues (N=6). **H**: The level of peripheral blood CD8^+^ Tex was significantly elevated following DMAb treatment compared to the level prior to treatment. **I**: The level of peripheral blood CD8^+^ Tcm was not elevated following DMAb treatment compared to the level prior to treatment. **J**: Kaplan-Meier curve revealed that patients treated with DMAb achieved a significantly longer median progression-free survival (PFS) compared to those treated with bisphosphonates (BPs) during anti-PD-1 immunotherapy. **K**: Multivariate analysis revealed that DMAb treatment was an independent protective factor for PFS in patients with LC-BoM undergoing anti-PD-1 immunotherapy, after adjusting age, sex, smoking status, performance status (PS) score, BoM burden, organ metastasis and histological subtypes. **L**: Kaplan-Meier curve revealed that patients treated with DMAb achieved a significantly longer median overall survival (OS) compared to those treated with BPs during anti-PD-1 immunotherapy. **M**: Multivariate analysis revealed that DMAb treatment was an independent protective factor for OS in patients with LC-BoM undergoing anti-PD-1 immunotherapy, after adjusting age, sex, smoking status, performance status (PS) score, BoM burden, organ metastasis and histological subtypes.

Continuous sections of the LC-BoM tissues as detected in Figure 4G (no-DMAb-treated) and 4I (DMAb-treated) were prepared for mIF staining of T cell subsets. CD8^+^ Tex cells were identified by the co-expression of CD8 and PD-1. CD8^+^ Tcm cells were identified by the co-expression of CD8, CD45RO, and CCR7 (**Figure 7A and B**). DMAb treatment significantly increased CD8^+^ T cell infiltration in LC-BoM tissues (**Figure 7C**).

In terms of spatial distribution, in LC-BoM tissues without DMAb treatment, a distinct ring of MMP19^+^ TAMs was observed surrounding the tumor cell nests, which acts as a barrier to prevent CD8^+^ T cells from infiltrating into the inner regions of the tumor (**Figure 7D**). By contrast, in LC-BoM tissues treated with DMAb, DMAb treatment reduced the number of MMP19^+^ TAMs and broke the circular barrier of MMP19^+^ TAMs. CD8^+^ T cells were extensively distributed in the peritumoral region, surrounding the tumor nests (**Figure 7E**). Quantitative analysis revealed that DMAb treatment significantly increased the levels of CD8^+^ Tex (**Figure 7F**) and Tcm (**Figure 7G**) in LC-BoM tissues.

We further explored the impact of DMAb treatment on peripheral blood T cell subsets by paired comparison in patients with BoM from solid tumors. Fifteen patients with newly diagnosed, untreated BoM from solid tumors were prospectively enrolled at FUSCC (**Clinical cohort 2 at FUSCC,** total n= 15) (**Supplementary Table S3**). Blood samples were collected to evaluate T-cell subsets prior to DMAb treatment and within five days following DMAb treatment for each patient. The changes in T-cell subsets before and after DMAb treatment were paired-compared. DMAb treatment significantly increased the levels of peripheral blood CD8^+^ Tex (**Figure 7H**), while having no significant effect on CD8^+^ Tcm (**Figure 7I**).

In order to investigate the clinical efficacy of DMAb in combination with anti-PD1 immunotherapy, we retrospectively included 119 patients with NSCLC-BoM who were treated at FUSCC between March 2018 and June 2023 (**Clinical cohort 3 at FUSCC**, total n=119). Of the 119 patients, 63 patients were treated with DMAb while 56 were treated with bisphosphonates (BPs) during the anti-PD1 immunotherapy (**Supplementary Table S4**).

Patients treated with DMAb had a significantly better median PFS than those treated with BPs (10 *vs* 4 months, P<0.05, **Figure 7J**). During the course of anti-PD1 immunotherapy, treatment with DMAb was significantly associated with a reduced risk of disease progression (adjusted HR=0.407, 95% CI=0.255-0.648, P<0.0001, **Figure 7K**). Patients treated with DMAb had a significantly better median OS than those treated with BPs (22 *vs* 15 months, P<0.05, **Figure 7L**). During the course of anti-PD1 immunotherapy, treatment with DMAb was significantly associated with a reduced risk of mortality (adjusted HR=0.544, 95% CI=0.343-0.862, P=0.01, **Figure 7M**).

### 3.8 Cell communications among tumor cells, MMP19^+^ TAMs and CD8^+^ T cell subsets in LC

**Figure 8:**
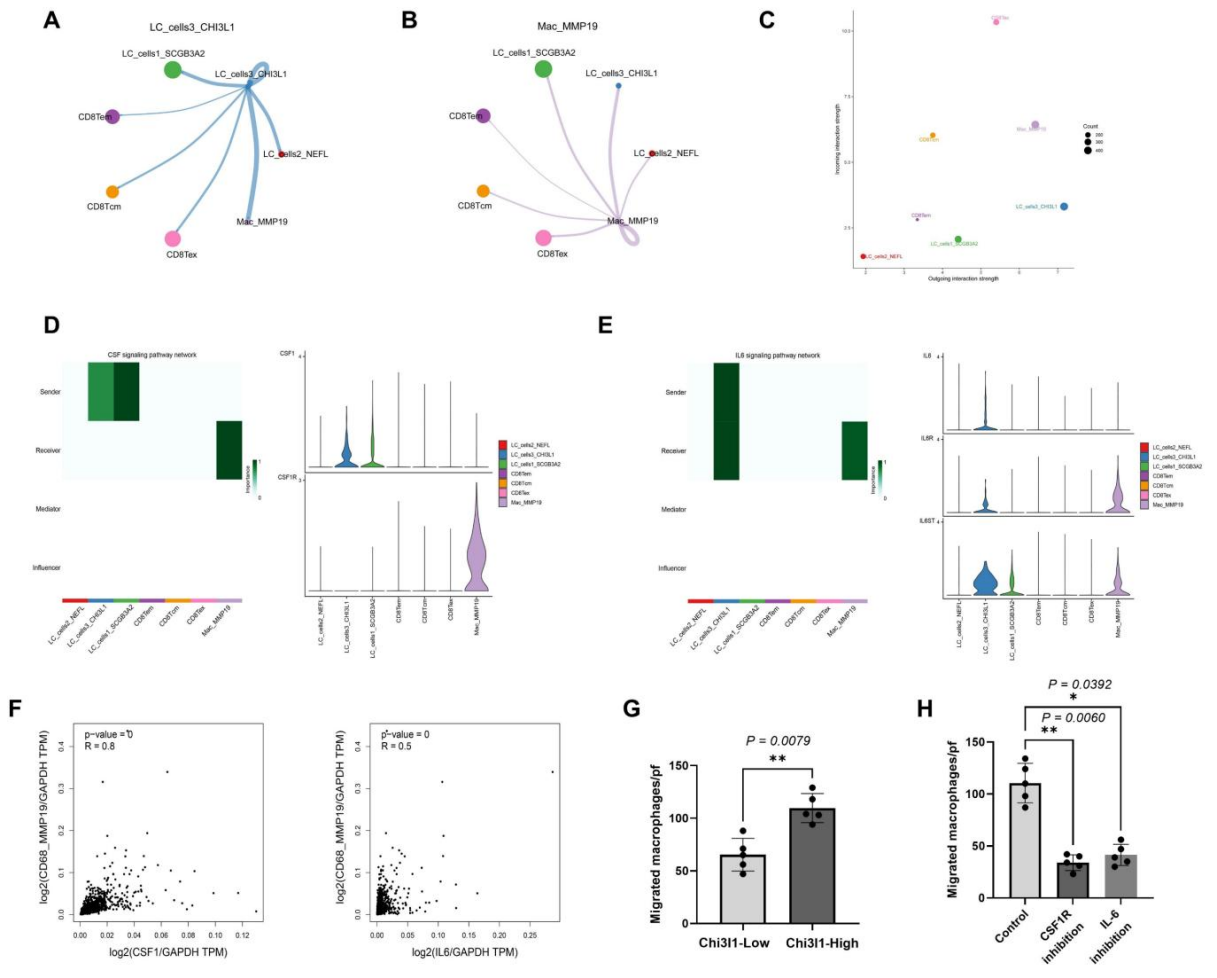
** Cell communications among tumor cells, MMP19^+^ TAMs and CD8^+^ T cell subsets in LC-BoM. A:** Output effects of LC_cells3_CHI3L1 on MMP19^+^ TAMs and CD8^+^ T cell subsets. The thicker the line, the greater the number of output effects. **B**: Output effects of MMP19^+^ TAMs on CD8^+^ T cell subsets and tumor cells. **C**: LC_cells3_CHI3L1 was the predominant sender with the strongest outgoing interaction strength, followed by MMP19^+^ TAMs. CD8^+^ Tex was the predominant receiver with the strongest incoming interaction strength, followed by MMP19^+^ TAMs. **D**: LC_cells3_CHI3L1 acted on MMP19^+^ TAMs via the ligand-receptor of CSF1 and CSF1R. LC_cells3_CHI3L1 as sender highly expressed CSF1, and MMP19^+^ TAMs as receiver highly expressed its receptor CSF1R. **E**: LC_cells3_CHI3L1 acted on MMP19^+^ TAMs via the ligand-receptor of IL-6 and IL-6R. LC_cells3_CHI3L1 as sender highly expressed IL-6, and MMP19^+^ TAMs as receiver highly expressed its receptor IL-6R. **F**: GEPIA2-derived database independently validated CSF1 and IL-6 were positively correlated with MMP19^+^ TAMs (CD68 and MMP19) expression. **G**: In the Transwell assay, LC cells with high expression of CHI3L1 recruited significantly more macrophages than those LC cells with low expression of CHI3L1 (P = 0.0079). **H**: In the Transwell assay, compared with the control group, the number of migrated macrophages in the CSF1R inhibitor group and the IL6 inhibitor group decreased significantly (P = 0.006 and P = 0.0392, respectively).

Based on the number of cell interactions, LC_cells3_CHI3L1 presented a strong output signal on Mac_MMP19 (**Figure 8A**). Mac_MMP19 presented output signals on CD8^+^ Tcm, Tem, and Tex (**Figure 8B**). LC_cells3_CHI3L1 exhibited the strongest outgoing interaction strength, followed by MMP19^+^ TAMs. CD8^+^ Tex exhibited the strongest incoming interaction strength, followed by MMP19^+^ TAMs (**Figure 8C**).

Comprehensive analysis of ligand-receptor interactions in the cell communications among tumor cells, MMP19^+^ TAMs and CD8^+^ T cell subsets was conducted. To be specific, in the interaction between LC_cells3_CHI3L1 and MMP19^+^ TAMs, LC_cells3_CHI3L1 secret colony-stimulating factor 1(CSF1), which subsequently interacts with the CSF1-receptor (CSF1R) expressed on MMP19^+^ TAMs (**Figure 8D**); LC_cells3_CHI3L1 may secret interleukin-6 (IL6), which subsequently interacts with the IL-6-receptor (IL6-R) expressed on MMP19^+^ TAMs (**Figure 8E**). In an independent validation using the transcriptomic data of LC available within GEPIA2, the genes CSF1 and IL-6 were positively correlated with MMP19^+^ TAMs expression level (**Figure 8F**). The Transwell assay was conducted *in vitro* to verify that the critical role of LC_cells3_CHI3L1 recruiting macrophages. In vitro, LC cells with high expression of CHI3L1 recruited significantly more macrophages than those LC cells with low expression of CHI3L1 (**Figure 8G**). The Transwell assay was also conducted to verify the critical role of the CSF1/CSF1R and IL-6/IL-6R ligand-receptor pairs in the process of LC cells recruiting macrophages. Intervention of the CSF1/CSF1R and IL-6/IL-6R ligand-receptor pairs significantly reduced the ability of CHI3L1 lung cancer cells to recruit macrophages (**Figure 8H**).

By contrast, the interaction between bone metastatic PC tumor cells and SPP1^+^ TAMs was mediated by the macrophage migration inhibitory factor (MIF) signaling pathway (**Supplementary Figure S4A**). The interaction between bone metastatic BC tumor cells and MMP19^+^ TAMs was mediated by the amyloid precursor protein (APP) signaling pathway (**Supplementary Figure S4B**).

To investigate the impact of MMP19^+^ TAMs on the function of CD8^+^ T cells, we extracted and cultured PBMC from 7 patients with LC-BoM and treated the PBMC with recombinant human MMP19 protein *in vitro*. Recombinant human MMP19 treatment significantly reduced the proportion of CD8^+^ T cells in lymphocytes (**Supplementary Figure S5 A**). Recombinant human MMP19 treatment significantly decreased the TNF-α level secreted by CD8^+^ T cells in the supernatant (**Supplementary Figure S5B**).

### 3.9 RANKL inhibitor combined with anti-PD1 immunotherapy in LC-BoM mouse model

**Figure 9:**
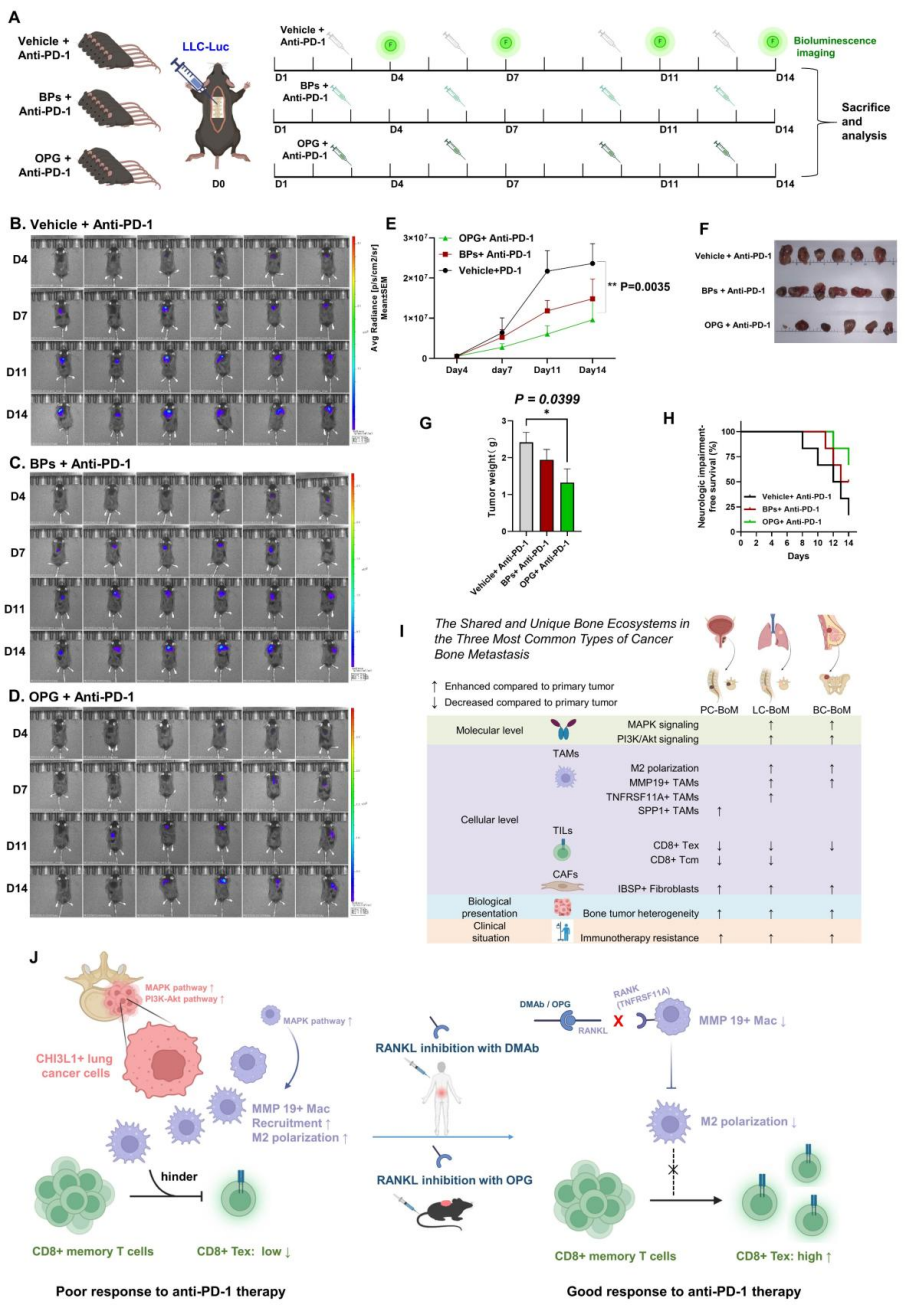
** RANKL inhibitor combined with anti-PD1 immunotherapy in LC-BoM mouse model. A**: Animal experiment design and work flowchart. A total of 18 mice with LC-BoM of the spine were randomly assigned to three groups: (1) Vehicle+ Anti-PD-1 (n=6); (2) BPs+ Anti-PD-1(n=6); and (3) OPG+ Anti-PD-1(n=6). **B**: Bioluminescence imaging for Vehicle+ Anti-PD-1 group on Day 4, Day 7, Day 11, and Day 14, respectively. **C**: Bioluminescence imaging for BPs+ Anti-PD-1 group on Day 4, Day 7, Day 11, and Day 14, respectively. **D**: Bioluminescence imaging for OPG+ Anti-PD-1 group on Day 4, Day 7, Day 11, and Day 14, respectively. **E**: The average fluorescence intensity of tumors in the OPG + Anti-PD-1 group was significantly lower compared to that in the Vehicle + Anti-PD-1 group on Day 14. **F and G**: The tumor weight in the OPG + Anti-PD-1 group was significantly lower than that in the Vehicle + Anti-PD-1 group. **H**: Kaplan-Meier curve showing the neurological impairments-free survival rate among the three groups. **I**: The summary of shared and unique bone ecosystems in the three most common types of cancer BoM. **J**: The schematic diagram showing the potential mechanism of RANKL inhibitor-induced immunosensitization.

We validated the synergistic effect of the RANKL inhibitor on anti-PD-1 immunotherapy in the LC-BoM mouse model. A total of 18 mice with LC-BoM of the spine were randomly allocated into three groups and subjected to corresponding drug treatments: (1) Vehicle+ Anti-PD-1 (n=6); (2) BPs+ Anti-PD-1(n=6); and (3) OPG+ Anti-PD-1(n=6) (**Figure 9A**). Bioluminescence imaging was conducted for each mouse in all groups on Day 4, Day 7, Day 11, and Day 14, respectively (**Figure 9B, C and D**).

The fluorescence intensity during the tumor growth process was markedly suppressed by the OPG combined with Anti-PD-1 treatment (**Figure 9E**). The combination of OPG and Anti-PD-1 treatment resulted in a significant reduction in the volume and weight of the tumors (**Figure 9F and G**). On the 14^th^ day, only 2 of the 6 mice (33.3%) in the OPG + Anti-PD-1 group exhibited spinal nerve-related impairments, a lower incidence compared to the BPs + Anti-PD-1 group (3/6, 50%) and the Vehicle + Anti-PD-1 group (5/6, 83.3%) (**Figure 9H**).

At the cellular level, increased MMP19^+^ TAMs and enhanced M2 polarization are critical factors contributing to BoM in LC and BC. Additionally, TNFRSF11A(RANK)^+^ TAMs represent a distinctive characteristic specific to LC-BoM. Regarding the immune microenvironment, a reduction in CD8^+^ Tex is consistently observed across PC-, LC-, and BC-BoM; whereas a reduction in CD8^+^ Tcm commonly occurs in PC- and LC-BoM. Increased intratumoral heterogeneity (ITH) within BoM are universal features observed in PC, LC and BC, which may underlie the resistance of BoM to immunotherapy (**Figure 9I**).

In LC-BoM, CHI3L1^+^ LC cells recruit and aggregate MMP19^+^ TAMs. These MMP19^+^ TAMs form a ring-shaped barrier structure surrounding the cancer cells, thereby inhibiting the infiltration of CD8^+^ T cells into the tumor interior and ultimately contributing to the resistance of LC-BoM to immunotherapy. RANKL inhibitors effectively decrease the number of MMP19^+^ TAMs and the M2 polarization level by targeting the receptor of RANK on the surface of MMP19^+^ TAMs. This treatment disrupts the ring barrier structure of MMP19^+^ TAMs, facilitating the infiltration of CD8^+^ T cells into the tumor interior, and consequently enhances the sensitivity of LC-BoM to immunotherapy (**Figure 9J**).

## 4. Discussion

The BoM microenvironment serves as the key regulatory system for tumor cells to metastasize to bones and form secondary lesions. A comprehensive analysis of both shared and distinct features of the BoM microenvironment across different cancer types is of great significance for understanding the mechanism of BoM and developing targeted treatment strategies 31. Key cellular components of the BoM microenvironment include tumor cells, bone marrow stromal cells (e.g., osteoclasts and osteoblasts), TAMs, immune cells, and endothelial cells 32. Our research findings demonstrate that the increase in myeloid cells represents a common characteristic of the microenvironment across cancer BoM.

During the process of BoM, are there particular types of tumor cells that exhibit a higher propensity for causing BoM compared to other tumor cells? In our study, we identified that PC_cells6, marked by the expression of PPAP2A, represents the predominant tumor cell subtype associated with PC-BoM. LC_cells3, marked by the expression of CHI3L1, constitutes the major tumor cell subtype associated with LC-BoM. Additionally, BC_cells3, marked by the expression of ESR1, serves as the primary tumor cell subtype associated with BC-BoM. All the three types of tumor cells associated with BoM exhibit high levels of ITH. ITH plays a crucial role in shaping the response pattern of immunotherapy by modulating the complexity of the tumor immune microenvironment. Tumors with high intratumoral heterogeneity (ITH) encompass heterogeneous regions, resulting in inconsistent treatment outcomes to immunotherapy. High ITH may result in T-cell exhaustion as a consequence of prolonged antigen exposure, thereby diminishing the efficacy and persistence of immunotherapy 33,34. Our research elucidates, from the perspective of ITH, the reasons for the poor responses of BoM to immunotherapy and identifies three predominant tumor cell subtypes responsible for high ITH.

TAMs play a critical role in facilitating cancer metastasis via multiple mechanisms, including the formation of pre-metastatic niches, remodeling of the tumor microenvironment, induction of immune suppression and escape, promotion of epithelial-mesenchymal transition, and execution of metabolic reprogramming 35,36,37. TAMs, especially the M2-type TAMs, promote cancer metastasis. During the invasion phase of the primary tumor, TAMs secrete matrix metalloproteinases (MMPs) (e.g., MMP-2, MMP-9, and MMP-13) to degrade the basement membrane and thus establish a pathway for cancer cell invasion. In the stage when tumor cells enter the bloodstream, TAMs secrete CXCL12 that facilitate tumor cell adhesion to the vascular endothelium, thereby evading clearance by the immune system. During the colonization phase at the metastatic site, TAMs secrete matrix metalloproteinases (e.g., MMP-9) and angiogenic factors within the metastatic tissue, further supporting the growth and expansion of the metastatic lesion 38,39,40. In our research, we identified a novel subtype of MMP19^+^RANK^+^ TAMs, which represents a factor promoting LC-BoM. Our findings suggest that MMP19^+^RANK^+^ TAMs may induce the differentiation and activation of osteoclasts by modulating relevant signaling pathways and facilitate the bone resorption.

In the tumor microenviroment, RANKL is secreted by various cell types, including tumor cells, osteoblasts and T cells. OPG, acting as a natural inhibitor of RANKL, competitively binds to RANKL, thereby effectively inhibiting the activation and maturation of osteoclasts. The dynamic balance between RANKL and OPG plays a critical role in regulating the homeostasis of bone metabolism. There is a severe imbalance in the RANKL/OPG ratio in the tumor microenvironment, characterized by overexpression of RANKL and insufficiency of OPG. Tumor cells or TAMs secrete a large amount of RANKL, while the expression of OPG in the tumor microenvironment is down-regulated, leading to excessive activation of osteoclasts 41,42. RANK-RANKL pathway is known to activate NF-kB and induce cell proliferation 43. We identified that in addition to osteoclasts, MMP19^+^ TAMs also exhibit high expression of RANK. This finding suggests that RANKL inhibitors may not only suppress osteoclast activity, which is the classical effect, but could also exert inhibitory effects on MMP19^+^ TAMs. In our study, MMP19^+^ RANK^+^ TAMs indeed upregulated the NF-kappa B signaling pathway and the MAPK signaling pathway.

TAMs represent a critical component of immunosuppressive cells within the tumor microenvironment and provide an "immune-privileged" microenvironment for tumor growth, invasion, and metastasis through multiple mechanisms. For example, TAMs-secreted IL-10 and TGF-β could inhibit the cytotoxic activity of CD8⁺ T cells and facilitate the expansion of regulatory T cells (Tregs). In addition, TAMs uptake glutamine, leading to the functional impairment of CD8⁺ cytotoxic T cells 44, 45, 46. Recent studies have increasingly indicated that TAMs are considered a promising target for enhancing cancer immunotherapy 47. For instance, a study has shown that targeting MARCO and IL37R on immunosuppressive TAMs in LC could inhibit regulatory T cell activity and enhance cytotoxic lymphocyte function 48. MMP-19 promotes tumor cell proliferation, invasion, epithelial-mesenchymal transition (EMT), angiogenesis and metastasis in the tumor microenviroment 49,50. Our research demonstrates that the RANKL inhibitor DMAb, which potentially blocks RANK on MMP19^+^ TAMs, reduces the number of MMP19^+^ TAMs, disrupts the circular barrier formed by MMP19^+^ TAMs, and consequently enhances the infiltration of CD8^+^ T cells into the core of LC-BoM lesions.

Both our single-cell and clinical cohort data suggest that the decreased proportions of CD8^+^ Tcm and CD8^+^ Tex represent critical immune features when LC develops BoM. Low levels of CD8^+^ Tcm and CD8^+^ Tex are associated with a poor prognosis of LC-BoM patients during immunotherapy. Terranova-Barberio *et al.* 51 have demonstrated that the immune characteristics of exhausted CD8^+^ T cells in peripheral blood and tumor samples may serve as potential biomarkers for predicting the efficacy of immune checkpoint inhibitors. Zhang *et al.* 52 drew a comparable conclusion in their study of patients with LC undergoing immunotherapy. They demonstrated that the infiltration patterns of CCR8+ regulatory T cells (Tregs) and CXCL13+ exhausted T cells (Tex) could serve as effective predictors of the immunotherapy response in patients with EGFR-mutated NSCLC. Compared with tumor tissue samples, immune markers in peripheral blood can be detected with high efficiency and reproducibility using flow cytometry. Our research demonstrates that peripheral blood CD8^+^ Tex and CD8^+^ Tcm have the potential to serve as reliable predictive biomarkers for assessing the efficacy of immunotherapy in patients with LC-BoM. The RANKL inhibitor DMAb treatment can elevate the levels of CD8+ Tex in both peripheral blood and tissues of patients with LC-BoM, predicting a better responsiveness to immunotherapy.

We observed in both clinical cohorts and mouse models that RANKL inhibitors enhance the efficacy of immunotherapy for treating LC-BoM more effectively than bisphosphonates. Asano et al. 53 and Li et al. 54 also reached a similar conclusion that combination therapy of immune checkpoint inhibitors with DMAb enhances clinical outcomes in NSCLC patients with BoM. DMAb demonstrates favorable synergistic effects when used in conjunction with immune checkpoint inhibitor treatment, thereby improving the management of BoM. Based on our findings, DMAb interferes with MMP19^+^RANK^+^ TAMs and increases the proportion of CD8^+^ T lymphocyte subsets, thereby exerting an immune-sensitizing effect.

## Conclusion

In conclusion, aiming at the three most prevalent types of bone metastatic cancers, this study provides a systematic summary of the shared and distinct characteristics of BoM microenvironment in comparison to their respective primary lesions. We have identified specific subtypes of tumor cells that may be associated with BoM in PC, LC, and BC. We identified a novel type of MMP19^+^ RANK^+^ TAMs which are involved in LC-BoM. These cells highly express RANK and can hinder the infiltration of CD8^+^ T cells into the BoM microenvironment. RANKL inhibitors can attenuate the immunosuppressive effect mediated by MMP19^+^RANK^+^ TAMs. These findings establish a robust experimental basis and provide compelling clinical evidence for the combination therapy of immunotherapy with DMAb in LC-BoM patients.

## Supplementary Material

Supplementary figures.

Supplementary table S1_Sample ID and data source.

Supplementary table S2. Clinical cohort 1 at FUSCC.

Supplementary table S3. Clinical cohort 2 at FUSCC.

Supplementary table S4. Clinical cohort 3 at FUSCC.
