# Supplementary material for: Pan-cancer bone metastasis atlas at single-cell resolution identifies a distinct tumor-associated macrophage subset for mediating Denosumab-induced immunosensitization in lung cancer bone metastasis
Source: Int J Biol Sci. 2026 Jan 1;22(1):365–86. doi: 10.7150/ijbs.119777 (PMC12681936; doi:10.7150/ijbs.119777)
Supplement: Supplementary file 1 — Supplementary figures. [file ijbsv22p0365s1.pdf]

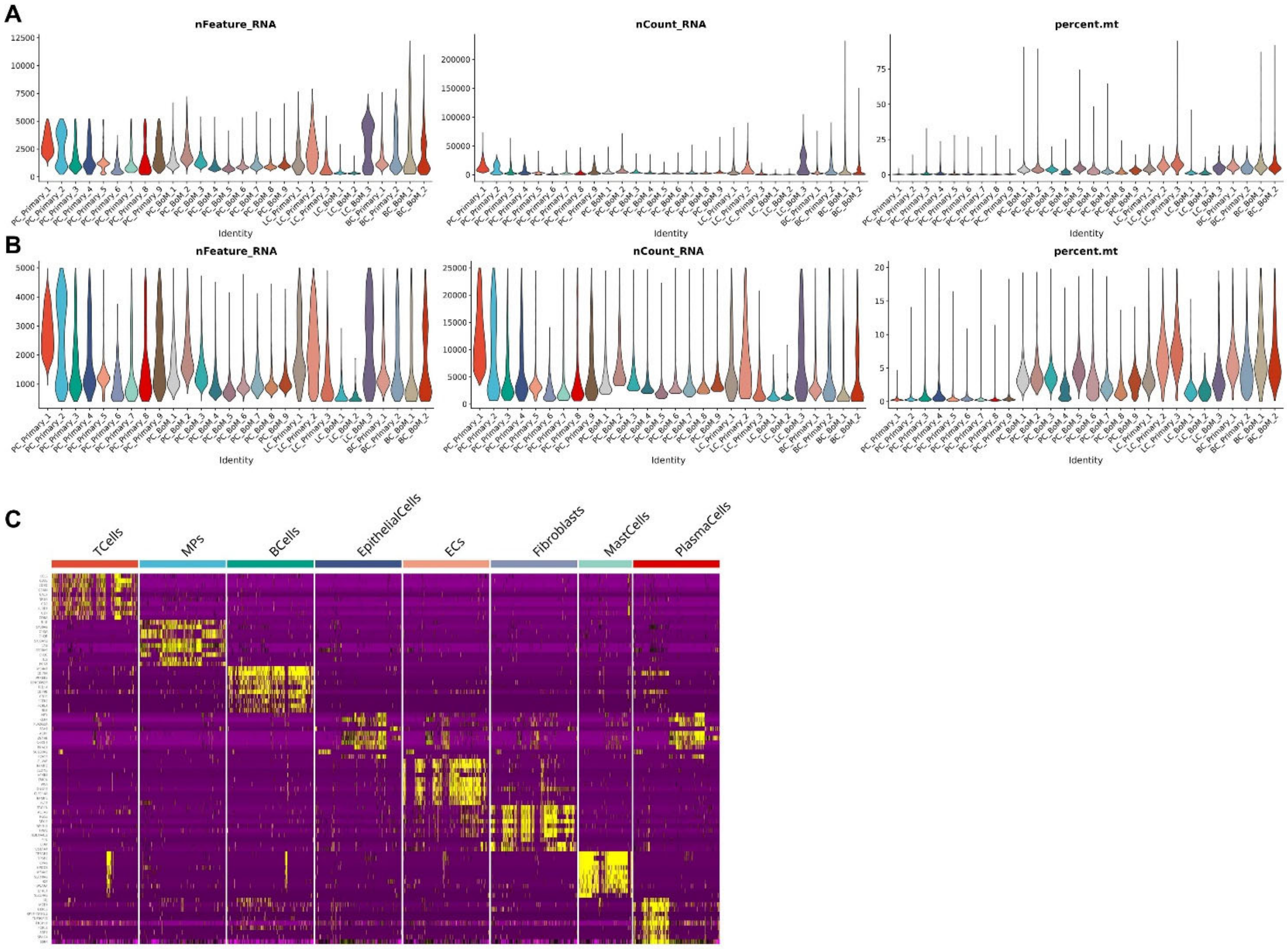

Supplementary Figure S1. Cell quality control, marker gene expression pattern of the eight cell subsets. (Related to Figure 1).

A: Before quality control, each sample's nFeature_RNA, nCount_RNA and percent. mitochondrial genes.

B: After quality control, each sample's nFeature_RNA, nCount_RNA and percent. mitochondrial genes.

C: Heatmap illustrating top ten highly expressed marker genes of each cell subsets.

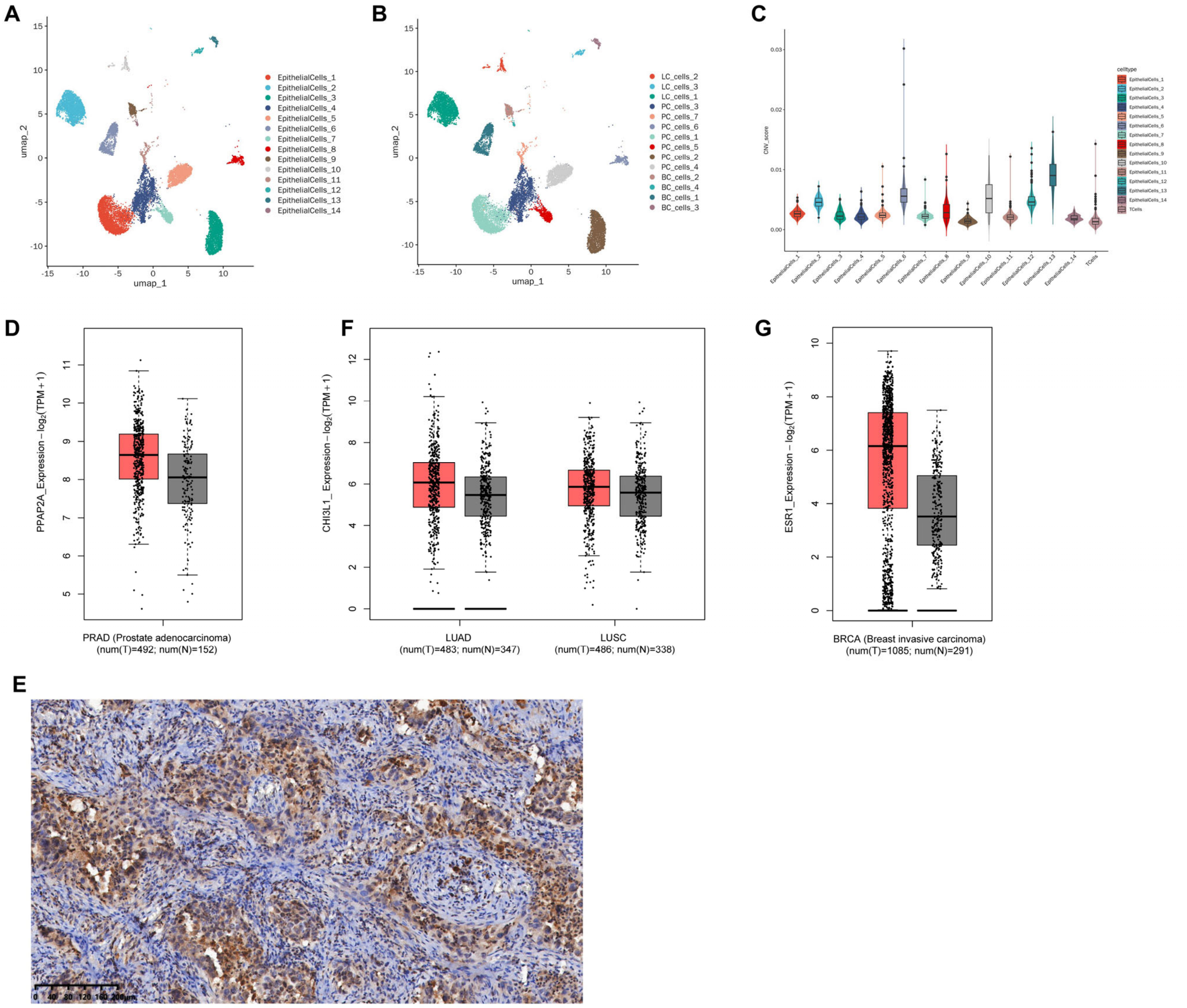

Supplementary Figure S2. Tumor cells subsets and marker genes expression. (Related to Figure 2)

A: UMAP plot showing the fourteen epithelial cells clusters.

B: UMAP plot shows that the 14 epithelial cells clusters were derived from 7 PC cells clusters, 3 LC cells clusters and 4 BC cells clusters.

C: Violin plot suggests that the fourteen epithelial cells clusters had elevated copy number variation (CNV) score compared to T cells.

D: The TCGA/GTEx data analysis validated that PPAP2A expression increased in prostate adenocarcinoma tissues compared to the normal prostate tissues,. T: tumor; N: normal.

E: Immumohistochemical (IHC) staining demonstrated that CHI3L1 expression in primary lung

adenocarcinoma (LUAD). CHI3L1 is moderately expressed in the cytoplasm of primary LAUD cells and barely in the tumor stroma. Scale = 40 μm.

F: The TCGA/GTEx data analysis validated that CHI3L1 expression increased in lung adenocarcinoma (LUAD) and lung squamous cell carcinoma (LUSC) tissues compared to the normal lung tissues. T: tumor; N: normal.

G: The TCGA/GTEx data analysis validated that ESR1 expression increased in breast invasive carcinoma tissues compared to the normal breast tissues. T: tumor; N: normal.

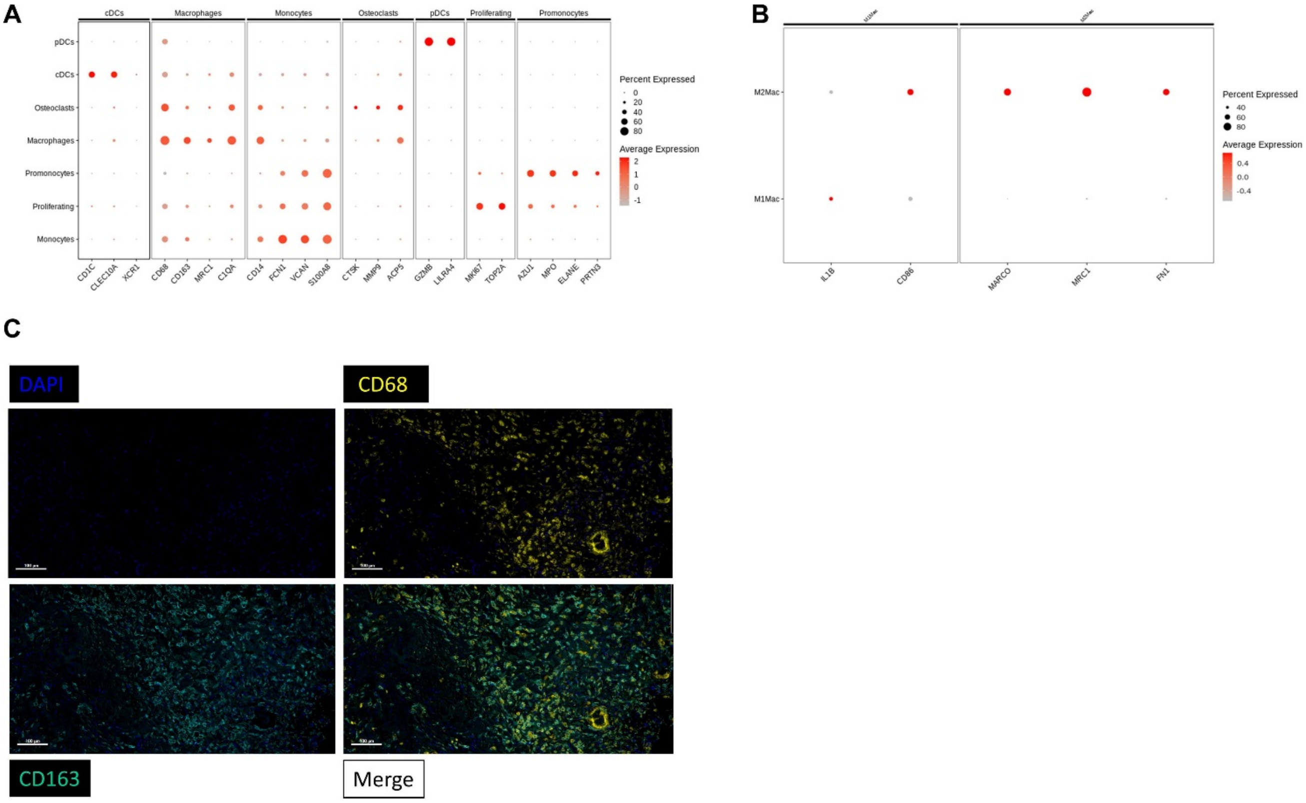

Supplementary Figure S3. Marker genes expression of the myeloid cells subsets **(Related to Figure 3).**

A: Dot plot showing the marker genes expression of the myeloid cells subsets.

B: Dot plot showing the marker genes expression of M1 and M2-TAMs.

C: Immunofluorescence staining further verified that M2-TAMs were widely expressed in LC-BoM tissues. TAMs were marked with CD68. M2-TAMs were further marked with CD163.

A

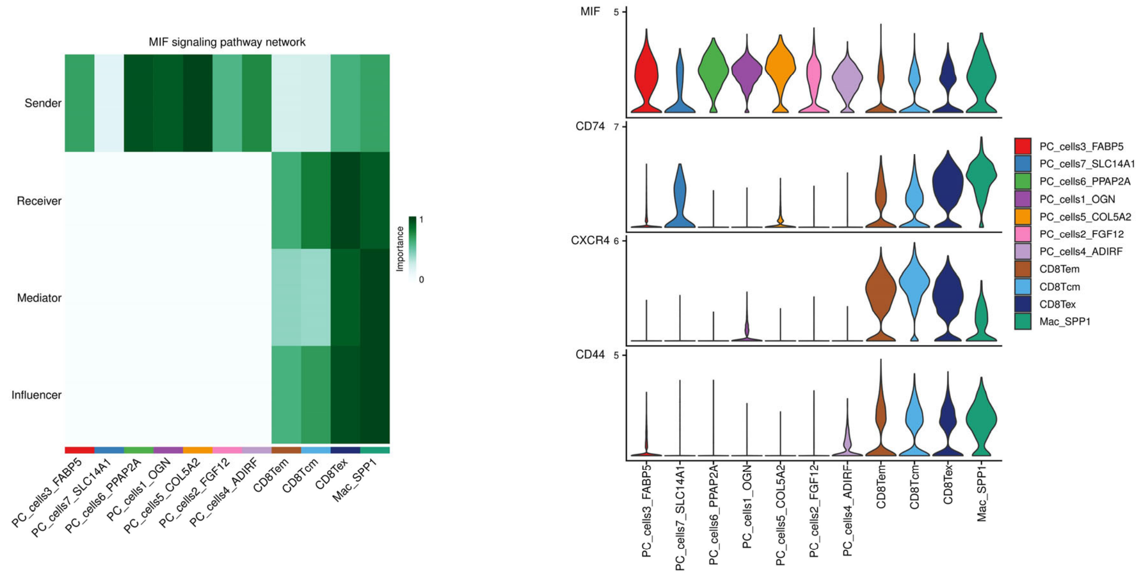

B

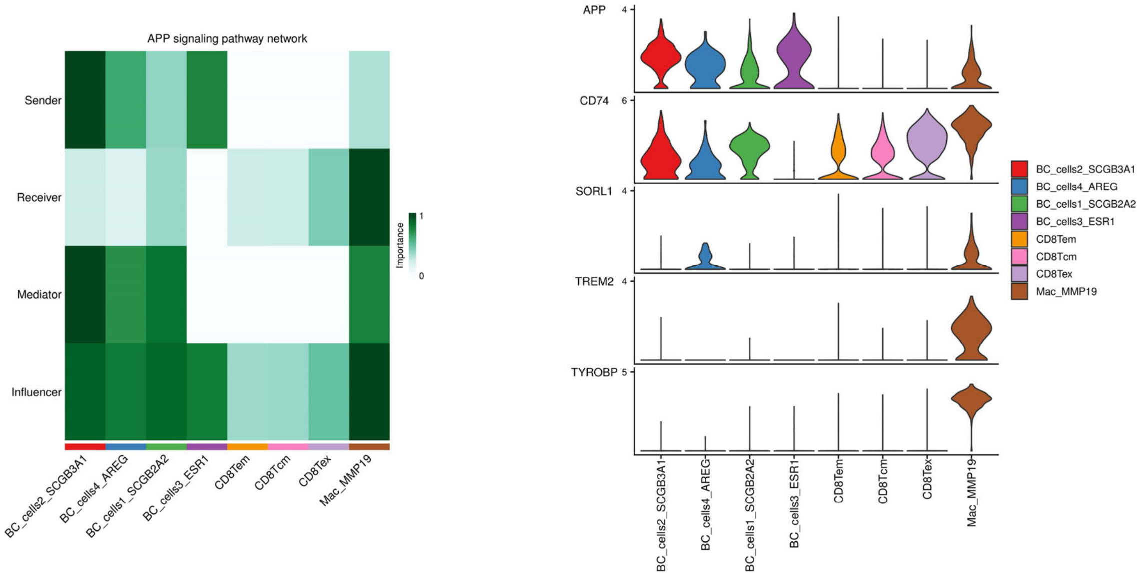

Supplementary Figure S4. Cell communications in prostate cancer (PC)-bone metastasis (BoM) and breast cancer (BC)-bone metastasis (BoM). **(Related to Figure 8).**

A: Bone metastatic PC tumor cells as senders highly express macrophage migration inhibitory factor (MIF). $SPP1^{+}$ TAMs as receiver highly express its receptors CD74 and CXCR4.

B: Bone metastatic BC tumor cells as senders highly express amyloid precursor protein (APP). $MMP19^{+}$ TAMs as receiver highly express its receptors CD74 and TREM2.

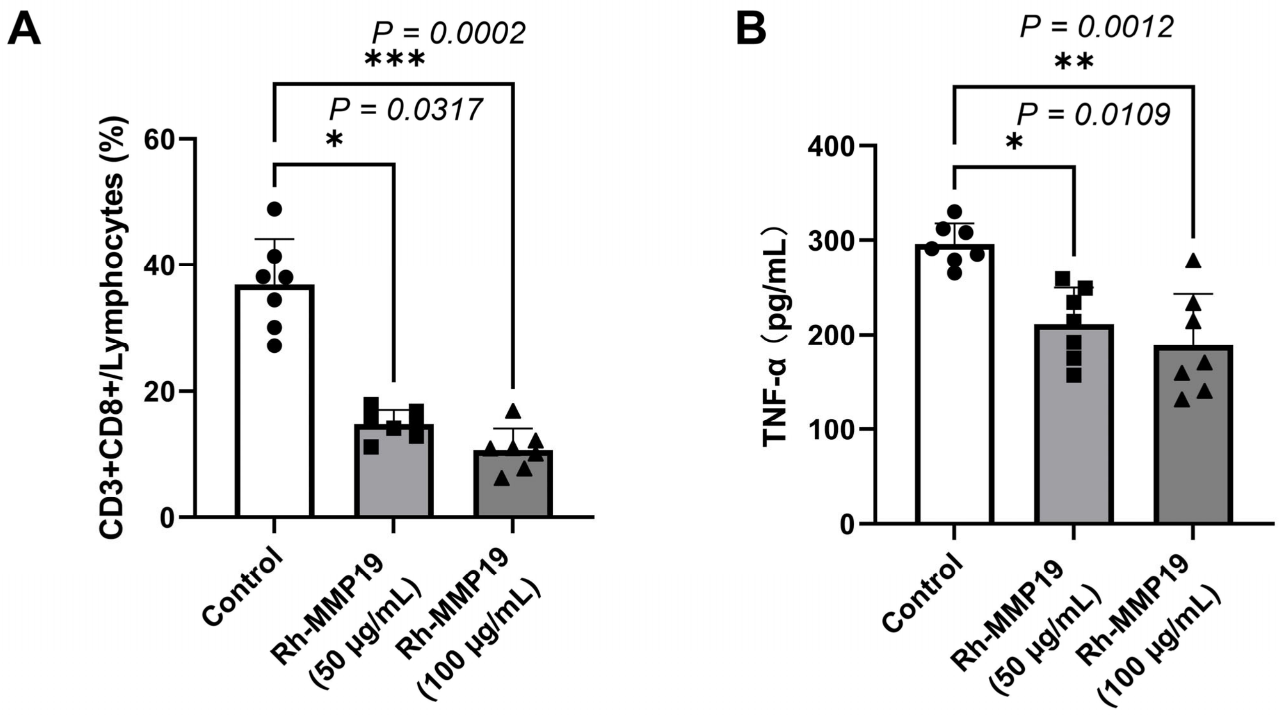

Supplementary Figure S5. The impact of MMP19 on the number and function of $CD8^+$ T cells **(Related to Figure 8).** Recombinant human MMP19 reduced the proportion of $CD8^+$ T (**A**). Recombinant human MMP19 decreased the TNF-α level secreted by $CD8^+$ T cells in the supernatant (**B**).
